# Strength is negatively associated with depression and accounts for some of the sex difference: A replication and extension

**DOI:** 10.1093/emph/eoac007

**Published:** 2022-02-22

**Authors:** Caroline B Smith, Tom Rosenström, Edward H Hagen

**Affiliations:** 1 Department of Anthropology, Washington State University, Pullman, WA, USA; 2 Department of Psychology and Logopedics, Faculty of Medicine, University of Helsinki, Helsinki, Finland

**Keywords:** mood disorders, major depressive disorder, bargaining, honest signaling, gender, replication

## Abstract

**Background:**

Depression occurs about twice as often in women as in men, a disparity that remains poorly understood. In a previous publication, Hagen and Rosenström predicted and found that grip strength, a highly sexually dimorphic index of physical formidability, mediated much of the effect of sex on depression. Striking results like this are more likely to be published than null results, potentially biasing the scientific record. It is therefore critical to replicate and extend them.

**Methodology:**

Using new data from the 2013–14 cycle of the National Health and Nutrition Examination Survey, a nationally representative sample of US households (*n* = 3650), we replicated models of the effect of sex and grip strength on depression reported in Hagen and Rosenström, along with additional potential confounds and a new detailed symptom-level exploration.

**Results:**

Overall, the effects from the original paper were reproduced although with smaller effect sizes. Grip strength mediated 38% of the effect of sex on depression, compared to 63% in Hagen and Rosenström. These results were extended with findings that grip strength had a stronger association with some depression symptoms, like suicidality, low interest and low mood than with other symptoms, like appetite changes.

**Conclusions:**

Grip strength is negatively associated with depression, especially its cognitive–affective symptoms, controlling for numerous possible confounds. Although many factors influence depression, few of these reliably occur cross-culturally in a sex-stratified manner and so are unlikely to explain the well-established, cross-cultural sex difference in depression. The sex difference in upper body strength occurs in all populations and is therefore a candidate evolutionary explanation for some of the sex difference in depression.

**Lay summary:** Why are women at twice the risk of developing depression as men? Depression typically occurs during social conflicts, such as physical or sexual abuse. Physically strong individuals can often single-handedly resolve conflicts in their favor, whereas physically weaker individuals often need help from others. We argue that depression is a credible cry for help. Because men generally have greater strength than women, we argue that men may be more likely to resolve conflicts using physical formidability and women to signal others for help. We find that higher grip strength is associated with lower depression, particularly symptoms like feeling down or thoughts of suicide and that strength accounts for part of the sex difference in rates of depression.

## DOES THE SEX DIFFERENCE IN PHYSICAL FORMIDABILITY EXPLAIN THE SEX DIFFERENCE IN DEPRESSION?

Depression is responsible for a lion’s share of non-fatal global disease burden [1], and women are about twice as likely as men to suffer depression. Hagen and Rosenström [2] found that among US adults (ages 18–60), grip strength largely mediated the sex difference in depression, controlling for potential risk factors for depression that also might vary by sex, including anthropometric, health, hormone and socioeconomic variables. Overall, the authors estimated that 63% of the total effect of sex on depression was mediated by grip strength.

These results have influenced research on, among other things, social inequality and gender disparities in mental health [3], the epidemiology, diagnosis and treatment of depressive disorders [4], health in aging adults [5] and the economic burden of inpatient care of depression [6]. Novel exciting results are more likely to be published than other important results with less publicity value, however, biasing the scientific record and misleading researchers and the public [7]. It is therefore important to replicate key findings with new data. Replication, however, could also inflate trust in findings that are misleading for reasons other than sampling noise and therefore ‘replicate’ well. To further test the underlying theory, it is important to include new elements in replication studies [8].

Here, we sketch the theoretical framework underlying the prediction that the sex difference in physical formidability explains the sex difference in depression [9]. We then replicate Hagen and Rosenström [2] using new data. We conduct exact replications when the same variables are available, and partial replications when variable substitutions are necessary, and we now control for additional possible confounds. To further investigate our theory, we also explore the associations of grip strength and sex on individual depression symptoms because, e.g. depression is likely heterogeneous, our theory predicts distinct functions of different symptoms [9], and risk factors for cognitive–affective symptoms differ from those for somatic symptoms [10, 11]. For example, when considering symptom-specific (independent) associations, inflammation is associated with the somatic but not the cognitive/affective symptoms of depression [11].

## COOPERATION, ADVERSITY AND CONFLICT

Humans are highly cooperative. Cooperation requires that the benefits outweigh the costs for *all* participants, however, and considerable conflict can arise when participants disagree on the divisions of benefits vs costs [12]. Conflict describes a state in which actions that increase the fitness benefit for one individual reduce the fitness benefit, or results in a fitness cost, for another. Conflict often arises in cooperative relationships, we argue, after one social partner experiences adversity, which we define as circumstances that have the potential to reduce an individual’s biological fitness. Adversity befalling one individual can cause conflict with her social partners because her attempt to mitigate her adversity and reduce her fitness cost often imposes costs on her social partners. Some examples of adversity include loss of a loved one, loss of a mate, illness or poor health, loss of resources and physical or sexual assault. In these cases, because the adversity is so severe, the victim is likely to require more support than he or she did before. If so, conflict might arise because meeting these new needs, such as providing care, resources, protection, emotional support or alloparenting, are costly for the social partners, some of whom could be unwilling or unable to increase their support (for review of the evidence, see [13]). Hagen [9] argued that both aggression and depression can serve as strategies to ‘bargain’ and to resolve conflicts with social partners in the wake of adversity.

## THE BARGAINING MODEL OF ANGER

Sell *et al*. [14] argue that anger is an adaptation to resolve conflicts in cooperative relationships in favor of the angry person. Specifically, because anger induces the angry individual to either withhold benefits or inflict costs on the target of their anger, anger functions to increase the importance the target puts on the angry person’s welfare relative to his or her own. Anger and formidability must be closely related because expressing anger without the capability to impose costs on the target could fail to change the target’s behavior, or even instigate retaliation. A range of studies, in both Western and non-Western samples, have found that anger is positively related to upper body strength and other indices of physical formidability [14, 15].

There is also evidence that neuroticism is negatively associated with physical formidability in Western samples [16]. Kerry and Murray [16] interpret neuroticism as heightened vigilance to threats, and less formidable individuals are more likely to benefit from higher vigilance toward potential threats in their environment. These findings suggest that physical formidability is one component of bargaining power, and as such it shapes anger, aggression and vigilance in adaptive ways.

## THE BARGAINING MODEL OF DEPRESSION

Adversity is a strong risk factor for major depression [17–19]. A number of researchers have proposed that low or depressed mood is an evolved response to adversity, with possible functions including energy conservation, risk avoidance, disengagement and problem analysis [20–26]. We test the bargaining model of depression [9]. Over human evolution, victims of adversity often needed help from social partners, and so signals of sadness such as facial expressions and crying have evolved to elicit support. Studies confirm that tears, for example, increase perceived sadness, sincerity and need for social support [27, 28]. When there is no conflict with social partners, these ‘cheap’ signals can effectively elicit help during times of adversity.

A little known but well-established fact about depression is that it is often closely associated with conflict and anger. Many of the major risk factors for depression *prima facie* involve conflict, such as intimate partner violence, physical and sexual assault, marital problems and divorce (for a review of the evidence, see [13]).

In order to receive help following adversity, when conflicts with social partners might cause them to be skeptical of cheap signals, victims must instead send a credible signal. According to the bargaining model, anhedonia, or loss of interest, a core feature of depression, can function as a credible signal of need, as can suicidality [9; see also 29]. In the game theory literature, strategies to resolve conflicts over the distribution of resources when the valuations of those resources are private information are studied as forms of bargaining (labor strikes are an example). A key finding is that (i) if parties can credibly signal their true valuations, agreement over the division of benefits can be reached immediately, but (ii) credibly signaling valuations often involves withholding cooperation for some period of time (which is costly because the value of the unused resources decreases with time [30]).

Hagen [9] argued that depression causes an individual to withhold her cooperation with social partners [see also 31], analogous to a labor strike, and that suicidality puts all future cooperation at risk, credibly signaling her need and thus eliciting greater support despite conflict. Unlike classic costly signals, in which there is a signaler and a receiver, in bargaining models, all parties can have private information that is revealed by their willingness to incur the costs of delay. Depressed people, by withdrawing from their economic and social sphere, or putting their lives at risk, are demonstrating that they are realizing so few benefits that they have nothing to lose by withdrawing (an option that is too costly for those whose lives are going well). Social partners, by helping or failing to help, also signal their high or low valuation, respectively, of the depressed people. In the labor strike analogy, for workers whose wages are genuinely too low, the benefits of signaling outweigh the cost of sacrificing their small wage, whereas for workers earning good wages, they do not. By the same token, employers signal their valuation of the workers by either quickly offering a raise (high valuation) or refusing to offer a raise (low valuation). All parties benefit when an agreement is reached [9, 31].

An experimental vignette study found that participants’ belief in a victim’s need, and their willingness to help the victim, increased monotonically with the cost of the victim’s signals, which ranged from crying (low cost) to depression (medium cost) to suicidality (high cost) [32].

## THE UNIFIED BARGAINING MODEL

Hagen [9] argued that both anger and depression can compel social partners to provide more help to victims of adversity. The Unified Bargaining Model [2] combines the bargaining theory of anger with the bargaining theory of depression. Physically formidable individuals can use anger and aggression to protect themselves from assault or exploitation, or otherwise resolve conflicts in their favor, whereas less physically formidable individuals might instead bargain with depression and suicidality.

### Sex differences in physical formidability and depression

Overall, humans are moderately sexually dimorphic, with a 7–8% difference in average height and 15% difference in average weight. Musculature and body strength, though, are highly dimorphic. On average, men have 61% more overall muscle mass and 78% more muscle mass in the upper arms. This concentrated muscle dimorphism in the arms and back translates to 90% greater upper body strength in men than women [33]. Using National Health and Nutrition Examination Survey (NHANES) data, we found that, in 95.7% of random encounters between a woman and man, the man would have higher grip strength. The sex difference in grip strength in these data was large (Cohen’s d = 2.5).

Depression is also sexually dimorphic: it is about twice as prevalent in women as men, with some cross-national variation [34]. The cause of this sex difference is unknown. Some have argued that since stressful life events precede depressive episodes, a sex difference in frequency or severity of stressful events could explain the sex difference in depression prevalence, although evidence has not shown a significant difference in either [35]. There is also not clear evidence that national variation in depression rates between the sexes is due to variation in gender equality [36].

Cross-sectional and longitudinal studies, mostly in older adults, have found a negative relationship between grip strength and depression (reviewed in [37]). Hagen and Rosenström [2] proposed that if depression is a strategy for less formidable individuals to resolve conflicts in their favor, then the sex difference in physical formidability might cause the sex difference in depression (see also [38]). They found that 63% of the effect of sex on depression was mediated by grip strength. Kerry and Murray [39] similarly found that controlling for strength reduced sex differences in trait anxiety.

## THE CURRENT STUDY

The aim of this paper is to replicate the analyses reported in Hagen and Rosenström [2] using new data. The original paper used publicly available data collected for the Centers for Disease Control NHANES during the 2011–12 survey cycle. Here, we use NHANES data from the 2013–14 collection cycle. NHANES uses a complex, multi-stage sampling strategy in order to collect data representative of the civilian, non-institutionalized United States population. NHANES combines interview, examination and laboratory data to assess health status and identify health risks for adults and children in the USA. Data collection occurs in new cycles every 2 years.

We refer to the 2011–12 cycle used by Hagen and Rosenström [2] as the Original dataset, the 2013–14 cycle analyzed here as the Replication dataset and 2011–14 together as the Combined dataset. The replication dataset was chosen for the study because it included grip strength, a main predictor variable (grip strength was not collected in subsequent cycles). Analyses were limited to adults aged 18–60 years because depression data were publicly available for this age group, and because strength is most stable during these years.

## METHODS

All measures were obtained from the NHANES 2011–2012 (G) and 2013–2014 (H) survey cycles (see Supplementary information for NHANES variable names).

### Outcome variables: depression and suicidal ideation

Depression was measured using the PHQ-9, a validated nine-item screening instrument for assessing depression severity [40]. Participants were asked to consider the past 2 weeks and assess how often they had been bothered by problems such as trouble sleeping, loss of interest in activities, feeling down or depressed, or change in appetite. Responses are scored from 0 (not at all) to 3 (nearly every day). Scores for each item are summed for a depression score ranging from 0 to 27. Depression outcome was also coded as a binary variable of depressed status, with a score of 10 or greater representing at least moderate depression [40]. Compared to diagnostic interview, a cutpoint of ≥ 10 on PHQ-9 substantially overestimates depression prevalence [41]. We maintain this cutpoint since it was used in the original study [2], and in order to generally discriminate very low depression scores from higher scores.

Suicidal ideation was coded as any nonzero response to the item ‘Thoughts that you would be better off dead or of hurting yourself in some way’ as indicative of suicidal ideation, although we note that ‘hurting yourself in some way’ might also indicate ideation of non-suicidal self-harm [42].

Because there is mounting evidence that depression is heterogeneous, and that specific depression symptoms are distinct phenomena that are influenced by specific life events, there are increasing calls to investigate depression symptoms individually rather than simply summing them [43]. We therefore also conduct an exploratory analysis in which we treat each of the nine PHQ-9 items as a separate outcome variable.

### Predictor variables

Grip strength, an index of upper body strength and physical formidability, was measured three times on each hand using a dynamometer. We used combined grip strength, which was the sum of the highest reading of each hand. All regression models included sex (male/female), age, grip strength and an age × strength interaction term, per the original paper, along with potential confounds described below [2].

### Potential confounds

We specified five regression models in order to determine if the effect of grip strength on depression was due to confounds with a range of anthropometric, socioeconomic, health and hormone variables that have been associated either with depression or with grip strength. We differentiate between exact and partial replications. Exact replications are those models specified in Hagen and Rosenström [2] for which the same variables were also collected in the 2013–14 cycle. Some variables were not repeated in the 2013–14 cycle, leading to our partial replications for which we included as many original variables as possible. We also extended the findings of Hagen and Rosenström [2] by including additional confounds in this paper.

#### Exact replications

We were able to exactly replicate the anthropometric and socioeconomic models. The anthropometric model included our main predictor variables plus standing height, weight, BMI ≥ 30 and an interaction term BMI ≥ 30: sex. The socioeconomic model included education level, whether the participant was living alone and Poverty Income Ratio (PIR). PIR was calculated as the ratio of family income to the local poverty threshold for each participant, controlling for family size (range 0–5, 0 indicates no income; 1 is income equal to the poverty threshold; values > 5 set to 5 to protect anonymity). In Hagen and Rosenström [2], the socioeconomic model did not include race, but since race is a proxy for disparities that affect depression prevalence, in the Supplementary information, we report the socioeconomic model with race included.

#### Partial replications

The hormone model included only serum total testosterone because thyroid-stimulating hormone and free thyroxine (T4 free) were not collected in the 2013–14 NHANES survey cycle.

The health model included white blood cell count as a control for infection and inflammation, as well as hemoglobin and perceived abnormal weight, but no longer included days of poor health, which was not measured, nor the physical disability score because we found it also included emotional disability (see the Supplementary information). We nevertheless needed to control for physical health conditions that might impact both depression and strength. For physical disability, we substituted a single item, ‘Special Equipment’ (PFQ054) (e.g. cane, walker), since a mental or emotional problem is unlikely to cause a need for special equipment to walk. For days of poor health, we substituted two variables: ‘Chronic disease score’ (CDS) and ‘Physical disease difficulty’ (PDD) since there is a bidirectional relationship between depression and chronic illness [44]. The CDS was a score (0–6) of chronic diseases including diabetes, cancer, stroke, arthritis, heart disease and respiratory disease (asthma, emphysema or chronic bronchitis). A point was added for each disease a participant indicated they had been diagnosed with, regardless of any impairment due to the disease [11]. PDD was calculated from a separate NHANES question, which asked participants to list up to five health conditions that specifically caused them to have difficulties with physical activities (0–5) (we excluded counts for ‘depression/anxiety/emotional problem’ and for ‘other impairment/problem’ because these conceptually overlapped with our depression outcome variable).

### Exploratory analysis of each symptom (conceptual replication)

To extend the original analysis [2], we explored if individual depression symptoms were differentially associated with sex and strength by fitting two models to each of the nine symptoms (18 models total). The first model included only sex and strength as predictors, and the second model included sex, strength and all the health variables as predictors because these were the predictors of depression that were most confounded with strength.

### Statistics

All analyses were completed in R version 4.0.4 (2021-02-15), using the survey package in order to incorporate the survey sampling weights and to preserve the representative structure of the sample. Mediation analyses were completed using unweighted data since the survey package cannot compute mediation analyses, and the mediation package cannot incorporate weights.

Regression models of depression score were computed using a Gaussian (normal) outcome distribution in order to replicate the models reported in Hagen and Rosenström [2]. However, depression scores are highly skewed and bounded on [0–27], with many values equal to zero. In Gaussian regression models of such outcome variables, the residuals are not normally distributed nor homoscedastic. For this reason, unlike [2], we additionally employed a common technique to model values on a closed interval by scaling our depression scores to 0–1 and then fitting a quasi-binomial model [45], which we report in the Supplement as a sensitivity test. Since the original paper reported models using Gaussian models, we use those in reporting our replications.

Individual depression symptoms are scored on a 0–3 point scale, and are skewed toward small values (mostly zeros). We also scaled these values to 0–1 and fit quasi-binomial regression models.

Continuous explanatory and control variables were centered at their means and divided by 2 standard deviations (SD) so that regression coefficients represent a 2 SD change, roughly from ‘low’ to ‘high’ values, and are directly comparable to those of binary variables with equal class probabilities, such as sex [46].

### Predictions

In Hagen and Rosenström [2], the regression coefficient of sex, which by itself was a significant positive predictor of depressed status and depression score, was no longer significantly different from zero after controlling for strength. Furthermore, strength was a significant negative predictor of depressed status and depression score. We predicted that these same effects would be replicated in the new Replication dataset. We also predicted that a new set of health confounds, including chronic diseases and difficulty imposed by chronic diseases, would not better explain the effect of sex on depression (i.e. that the protective effect of strength was not due to confounds with other health-related variables and its protective effect thereby might be attributable to strength’s evolved role in bargaining).

### Ethical statement

This research was certified as not Human Subjects Research by the Washington State University Institutional Review Board, and therefore did not require review.

## RESULTS

There were *n* = 4192 adult participants in the Original dataset, *n* = 4384 in the Replication dataset and *n* = 8576 in the Combined dataset. The number of observations varied slightly per model due to missing data or NHANES sampling strategies that targeted subpopulations. See Table 1 for summary statistics of continuous variables in the Replication dataset.

**Table 1.** Summary statistics for the Replication dataset

Variables	Females					Males					*d*
	*n*	Min	Max	Mean	SD	*n*	Min	Max	Mean	SD	
Age (years)	2286	18.0	60.0	38.9	12.4	2098	18.0	60.0	38.8	12.6	0.0090
Depressed	1979	0	1.00	0.118	0.323	1855	0	1.00	0.0513	0.221	0.24
Depression score (PHQ-9)	1979	0	27.0	3.81	4.71	1855	0	27.0	2.39	3.59	0.34
Grip strength (kg)	2041	17.6	102	59.5	10.4	1915	21.9	163	94.0	16.7	−2.5
Height (cm)	2198	141	186	163	6.76	2014	147	203	176	7.56	−1.9
Weight (kg)	2195	35.6	202	77.9	22.6	2016	32.8	223	89.1	21.4	−0.51
Perceived abnormal weight	2283	0	1.00	0.646	0.478	2093	0	1.00	0.529	0.499	0.24
Physical disability score (0–14)	2100	0	14.0	1.15	2.88	1940	0	14.0	0.668	2.18	0.19
Special equipment	2100	0	1.00	0.0485	0.215	1940	0	1.00	0.0326	0.178	0.080
White blood cell count (1000 cells/µl)	2136	2.70	26.1	7.65	2.39	1944	2.50	31.4	7.32	2.25	0.15
Hemoglobin (g/dl)	2136	7.60	18.5	13.3	1.20	1944	6.40	19.5	15.2	1.11	−1.6
Testosterone (ng/dl)	2102	1.05	575	26.4	25.4	1916	55.1	1,550	423	169	−3.3
Poverty Income Ratio (0–5)	2109	0	5.00	2.76	1.71	1922	0	5.00	2.94	1.69	−0.10
Education level	2284	1.00	5.00	3.76	1.09	2098	1.00	5.00	3.66	1.15	0.089
Living alone	2286	0	1.00	0.0830	0.276	2098	0	1.00	0.0965	0.295	−0.047
Chronic disease score (0–6)	2092	0	5.00	0.596	0.844	1928	0	5.00	0.432	0.675	0.21
Physical disease difficulty (0–5)	2286	0	5.00	0.365	0.972	2098	0	5.00	0.226	0.786	0.16

For each of the four models with potential confounders (Anthropometric, Socioeconomic, Hormone and Health), we include a plot of the coefficients for both depressed status and depression score, for models fit on the Original, Replication and Combined data.

### Exact replications

**Figure 1.**
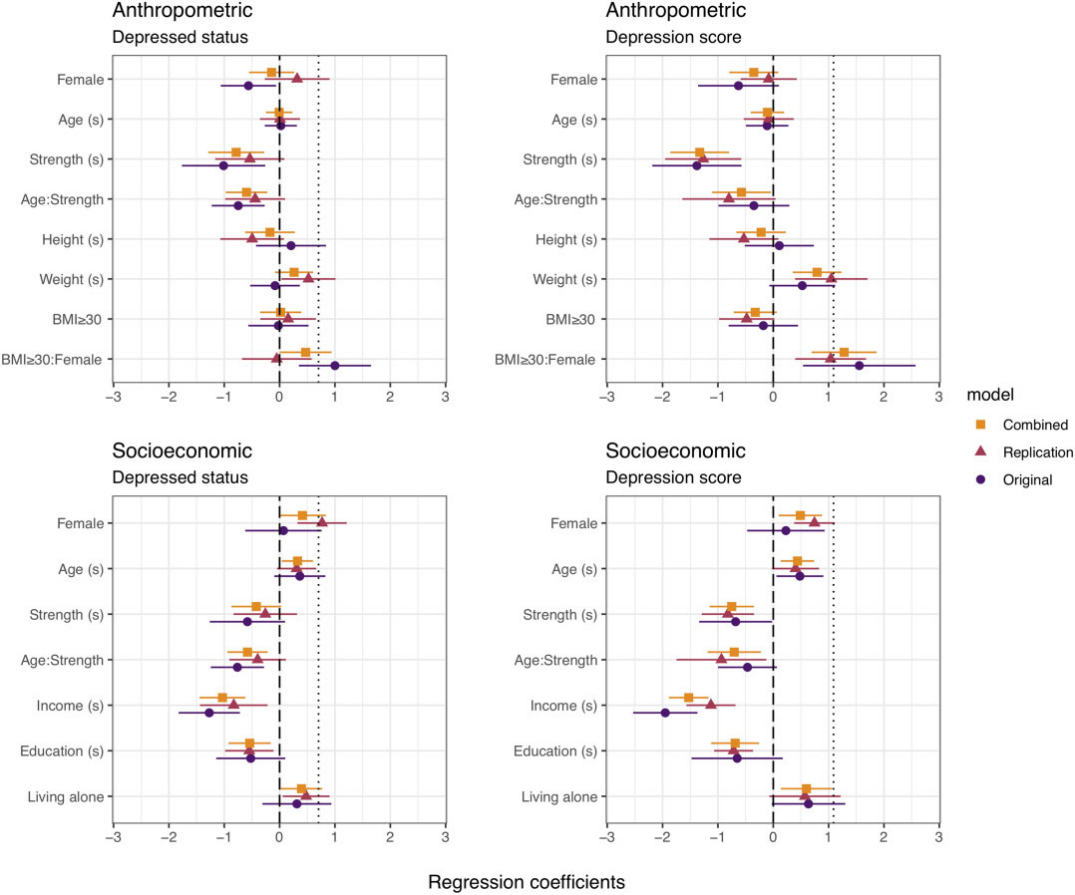
Coefficient plot for Anthropometric and Socioeconomic models of Depressed Status and Depression Score. Variables with (s) have been centered at their means and standardized by 2 SD. Dotted line marks the coefficient of sex alone

**Figure 2.**
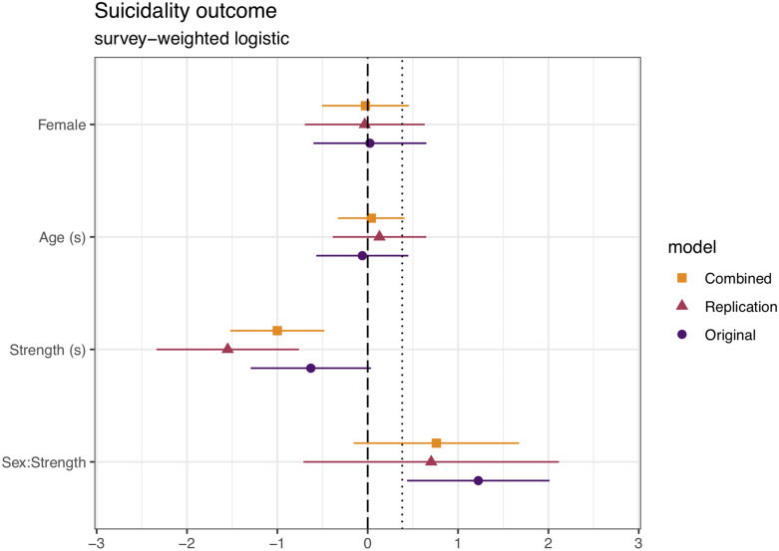
Coefficient plot for model of the Suicidal outcome. Variables with (s) have been centered and standardized by 2 SD

Figure 1 shows the coefficients for the exact replication models (the anthropometric and socioeconomic models) fit on the original data, replication data and combined data, and Fig. 2 shows the suicidality model. The coefficient of sex alone is shown by the dotted line. In the replication anthropometric models of depression status and score, and in the socioeconomic model of depression score, the coefficient of sex was reduced compared to sex alone, as predicted, but was larger than in the original models, contrary to predictions. However, the increase in the sex coefficient in the replication vs original data series was not statistically significant (see Supplementary Table S17).

In the socioeconomic model of depressed status, the coefficient of sex was not reduced relative to sex alone, contrary to predictions. However, the difference in sex coefficient between the original and replication datasets was only statistically significant for depression score (see Supplementary Table S17).

In all replication models, the coefficients of strength and/or the age: strength interaction, were negative, as predicted, albeit not statistically significantly so in the anthropometric and socioeconomic model of depressed status, contrary to predictions. In the combined data (2011–14), the coefficients of sex, strength and age: strength interaction were intermediate between those in models fit on the original vs replication data.

In the original dataset, the interaction term BMI ≥ 30: sex was a significant predictor of depression status and score, but only for depression score in the replication dataset. Race was not included in the original socioeconomic model. However, race reflects patterns of access to resources and exposure to structural violence. Adding race to the socioeconomic model did not substantially alter the sex and strength coefficients. See Supplementary Tables S1–S6 in the Supplementary information for model parameters.

### Partial replications

**Figure 3.**
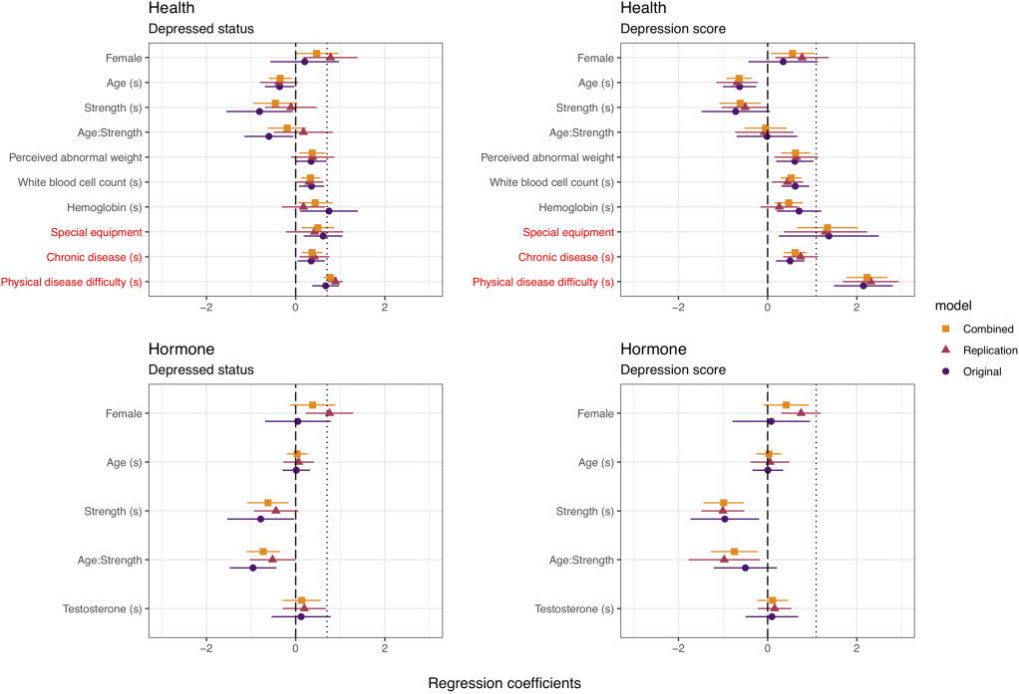
Coefficient plot for Health and Hormone Models of Depressed Status and Depression Score. Variables with (s) have been centered at their means and standardized by 2 SD. Dotted line marks the effect of sex alone. Variables listed in red were not included in the original models

The health and hormone model coefficients are shown in Fig. 3. In the replication dataset, the coefficient of sex was reduced relative to sex alone in the models of depression score, as predicted, but not the models of depressed status, contrary to predictions. Furthermore, in the partial replication models fit on the replication data, the 95% CI of sex overlaps with the point estimate of sex alone, contrary to predictions. In all models, the coefficient of sex was larger in the replication compared to the original models, contrary to predictions. Similar to the exact models, then, the addition of grip strength does not reduce the effect of sex to the same degree as it did in the original dataset (but see quasi-binomial coefficients in Supplementary Fig. S4). In the combined dataset (2011–14), the effect of sex was intermediate between the original and replication datasets. See Supplementary Tables S7–S12 in the Supplementary information for model outputs.

### Mediation analysis

We estimated the proportion of the sex effect on depression that is mediated by grip strength under causal mediation. We used a moderated mediation that modeled grip strength with age and sex in the mediation model. This allowed for the proportion of the sex effect mediated to vary with age. In the original dataset, we found that 63% of the total effect of sex on depression was mediated by strength.

In this non-weighted analysis using the replication dataset (2013–14), we estimated that female sex increased depression prevalence by 6.4 percentage units compared to men (5.5% prevalence), and altogether 39% of that total effect of sex on depression was mediated by grip strength (CI = 5.2–77% and *P* = 0.026 for mediated effect).

**Figure 4.**
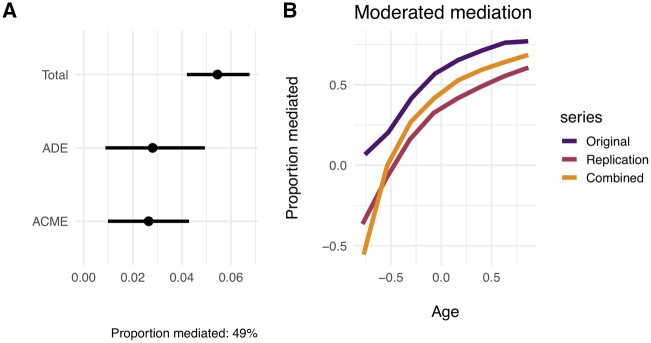
Mediation of sex effect on depression by strength in the combined dataset. (**A**) Average mediation effects. Estimated mediation effects were similar for both men (open circles; dotted line for 95% confidence interval) and women (closed circles; solid line for 95% confidence interval). (**B**) Moderated mediation. Estimated proportion of the total sex effect that is mediated by strength, given as a function of age (the moderating variable). Age is centered and standardized by 2 SD. ACME = Average Causal Mediation Effect; ADE = Average Direct Effect. Notice that Total Effect is ACME + ADE, averaged over sexes, or ‘treatments’. Proportion mediated is average ACME divided by the Total Effect; interpretation of the proportion is straightforward only when ACME and ADE are of the same sign

In the combined dataset (2011–14), we estimated that female sex increased depression prevalence by 5.5 percentage units compared to men (6.1% prevalence), and altogether 48% of that total effect of sex on depression was mediated by grip strength (CI = 18–83% and *P* < 0.001) for mediated effect (see Fig. 4).

### Explaining differences in the original vs replication survey cycles

There was one difference in the two survey cycles that might partially explain the differences in the sex coefficients in models fit on these two datasets. While mean depression scores were similar between cycles, there was a significant interaction between sex and survey cycle, such that the effect of female sex on depression was larger in the replication data but its effect on strength was similar to the original data. See Supplementary Table S16 and Fig. S5.

### Exploratory: associations of sex and strength with individual depression symptoms

**Figure 5.**
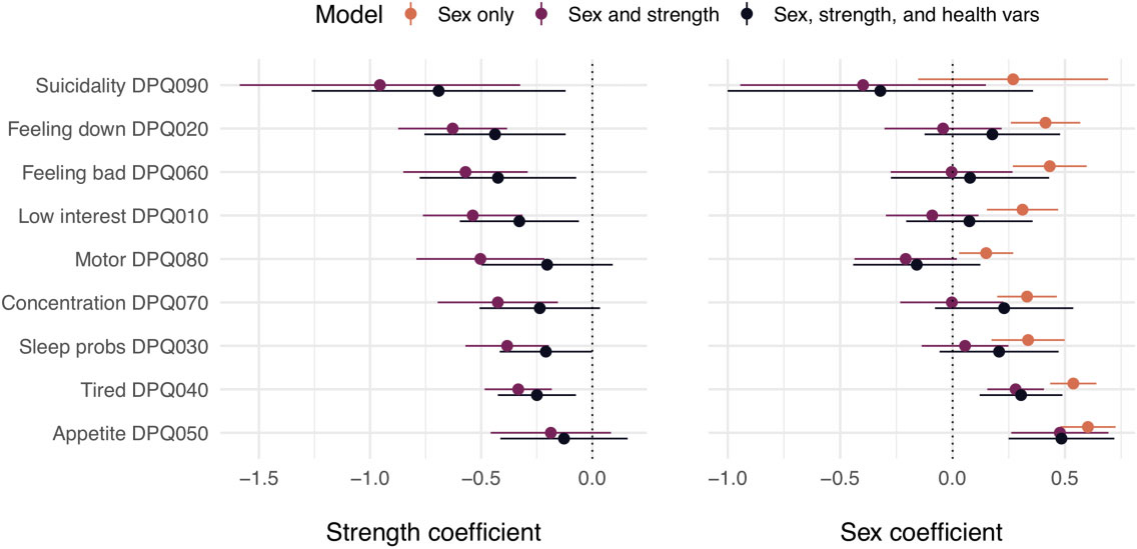
Coefficients of quasibinomial regression models of each symptom as functions of sex; sex and strength; and sex, strength, and the potentially confounding health variables. Coefficients ordered from negative to positive

In models of individual depression symptoms that included only sex and strength, the sex coefficient was only statistically significant (and positive) for appetite changes and tired or little energy (Fig. 5, left). The same pattern for sex was seen in models that also controlled for the health variables. Strength was most strongly and significantly negatively associated with the affective depression symptoms, whether controlling for sex alone or also controlling for the health variables, which included white blood cell count, a biomarker of inflammation (Fig. 5, right).

### Reverse causation

It could be the case that depression causes lower grip strength, e.g. because depressed individuals do not exert maximal effort in the grip strength test. NHANES includes a dichotomous variable that indicates whether the participant exerted a maximal or questionable effort during the grip strength test, as assessed by the technician. Hagen and Rosenström [2] found no significant association between depressed status and questionable effort. In the replication data, however, there was a significant positive association by a Chi-square test, *P* = 0.022, raising the possibility that depression causes low grip strength. We addressed this possibility using the Replication and Combined data sets. First, we removed the *n* = 42 individuals exerting questionable effort (0.49% of the participants), and then refit all models. There were negligible differences in the strength coefficients (see Supplementary Fig. S2).

## DISCUSSION

Due to biased publication, it is common that effect sizes are reduced in replication studies [47], and that was the trend here. We found that strength mediated a smaller percentage of the sex difference in the replication data than it did in the original data (62% in the original data, 39% in the replication data, 48% in the combined data). The protective effect of strength was also smaller. Nevertheless, the inclusion of grip strength in the models reduced the effect of sex on depression by about half, although there was variation among the models. Although coefficients for strength are smaller in absolute magnitude compared to those reported in Hagen and Rosenström [2], they are still statistically significant in most (but not all) models after controlling for a wide range of confounds, suggesting strength has an independent protective effect on depression.

The differences in the effect sizes between the two datasets might be partially explained by the larger effect of sex on depression in the replication data. The cause(s) of this increased effect are unclear. Even though both samples are representative of the US population, there is still sampling error. There could also have been shifts in exposure to adversity in women vs men. In this time frame (2011–15), social media use was intensifying, and there is evidence that social media use has a stronger association with depression in women than men, at least in adolescents and young adults [48]. We also investigated if sex differences in socioeconomic variables, including income, education, partner status, healthcare, and food insecurity differed by NHANES series and, if so, whether controlling for those variables would account for the increased effect of sex, but they did not (results not reported).

Depression is heterogeneous and specific depression symptoms are distinct phenomena that are influenced by specific life events [43]. Our exploratory analyses of individual depression symptoms, which controlled for a biomarker of inflammation and other health variables, found that strength had the most protective effect against symptoms such as suicidality, low interest, feeling down, and feeling bad. A diagnosis of a major depressive episode requires loss of interest or sad or low mood, so these symptoms are arguably core to depression. Because low interest and suicidality reduce one’s contribution to cooperative endeavors, or put it at risk, and because low mood often involves signaling, such as sad facial expressions or crying, these symptoms are also central to the bargaining model. For these symptoms, the coefficient of sex was not statistically significant after controlling for strength, indicating that the sex difference in strength might explain the sex difference in these core depression symptoms.

It is important to reiterate that our aim was only to explain the *sex difference* in depression, not all risk factors for depression. Many instances of adversity do not involve conflict, and under our theory therefore do not require bargaining. Death of a beloved family member might cause intense sadness, low mood and disrupt sleep and concentration exceeding the threshold for depressed status, for example, but if there were no conflict with social partners—e.g. all were highly supportive—sex differences in formidability would not result in sex differences in depressive symptoms. Our claim is that the sex difference in depression arises because adversity often does involve conflict, and the female disadvantage in physical formidability therefore increases the likelihood that females will attempt to resolve conflicts in their favor via depression and males with anger.

There are forms of adversity and conflict that cannot be resolved by signaling or threatening close social partners. These include structural conflict involving race, class or gender disparities in systems with marked power dynamics, such as healthcare, education, financial and legal systems. Rosenström [31] introduced a variant bargaining model of depression as ‘not participating’ in an ongoing collective action that might better account for depressive symptoms in these situations.

In summary, many factors can influence risk of depression and differentially affect men and women, but not many reliably occur in the same way across cultures. Such reliably occurring factors, like the sex difference in physical formidability, might therefore be important in explaining the well-established cross-cultural sex difference in depression prevalence.

### Limitations

Our study utilized cross-sectional datasets, meaning we are unable to determine causation based on temporal precedence. It is possible that depression causes lower grip strength, for instance (but removing participants with questionable effort on grip strength did not substantively alter our results), or that there are unmeasured confounds. For example, an alternative evolutionary account of depression predicts reallocations of energy from muscles to brain to support rumination [21, 49]. Furthermore, hand grip strength is only a proxy for overall formidability, and in some cases may not accurately reflect overall ability to bargain through aggression (for instance, a person who uses a wheelchair may have a high grip strength, or a person with low grip strength may have formidable allies).

Importantly these results suggest that depression is highly sensitive to environment. NHANES focuses on physiological health risk factors, and does not measure behavior, social support, adversity, conflict or stress. As a result, analyses involving depression using NHANES data are often de-contextualized from a person’s experiences, and models specified while missing these variables cannot fully address depression risk factors.

## CONCLUSION

The popular conceptualization of depression as a brain disorder has not resulted in improved treatment options or outcomes [50]. We argue it is important to consider physical factors such as formidability (e.g. body size, upper body strength) that might shape interactions with social partners and thus also shape risk of depression—especially symptoms like suicidality, low interest, feeling down and feeling bad. As this replication study has shown, although effects sizes were smaller than in Hagen and Rosenström [2], physical formidability might protect against depression, and sexual dimorphism in upper body strength (proxied here with grip strength) may partially account for the sex difference in depression.

## SUPPLEMENTARY DATA

Supplementary data is available at *EMPH* online.

## FUNDING

T.R. was supported by the Academy of Finland (grant numbers 334057 and 335901). The funder had no role in the manuscript preparation nor decision to publish.

**Conflict of interest:** None declared.

## Supplementary Material

eoac007_Supplementary_Data
