# Supplementary material for: Strength is negatively associated with depression and accounts for some of the sex difference: A replication and extension
Source: Evol Med Public Health. 2022 Feb 22;10(1):130–41. doi: 10.1093/emph/eoac007 (PMC8935202; doi:10.1093/emph/eoac007)
Supplement: eoac007_Supplementary_Data [file eoac007_supplementary_data.docx]

# Supplementary information

## Additional methods

Disability Score (pfq file; see code for this calculation) was calculated based on participant self-reported difficulty with performing physical activities that affected their ability to work. Participants were read the following script: “The next questions ask about difficulties you may have doing certain activities because of a health problem. By “health problem” we mean any long-term physical, mental or emotional problem or illness (not including pregnancy). By yourself and without using any special equipment, how much difficulty do you have [performing X task].” Some examples of tasks that were included in the measure are: “walking for a quarter of a mile,” “lifting or carrying,” “doing chores around the house.” Participants then rated the difficulty they had with each task on the following scale: “No difficulty,” “Some difficulty,” “Much difficulty,” “Unable to do,” “Do not do this activity.”

## Disability score

We discovered that the disability score variable included in the original paper is confounded with depression because the instructions directed participants to consider difficulty in a range of activities caused by “any long-term physical, mental or emotional problem or illness, not including pregnancy.” Since disability score (included on the right-hand side of the regression equation) included mental or emotional problems, it overlaps with depression (on the left-hand side of the regression equation); these models are therefore likely misleading. We nevertheless chose to replicate them since our primary goal in this paper was to replicate the original models as closely as possible. The disability model reported here includes the same variables reported in the health model in Hagen and Rosenström [1], with the exception of number of days of poor health.


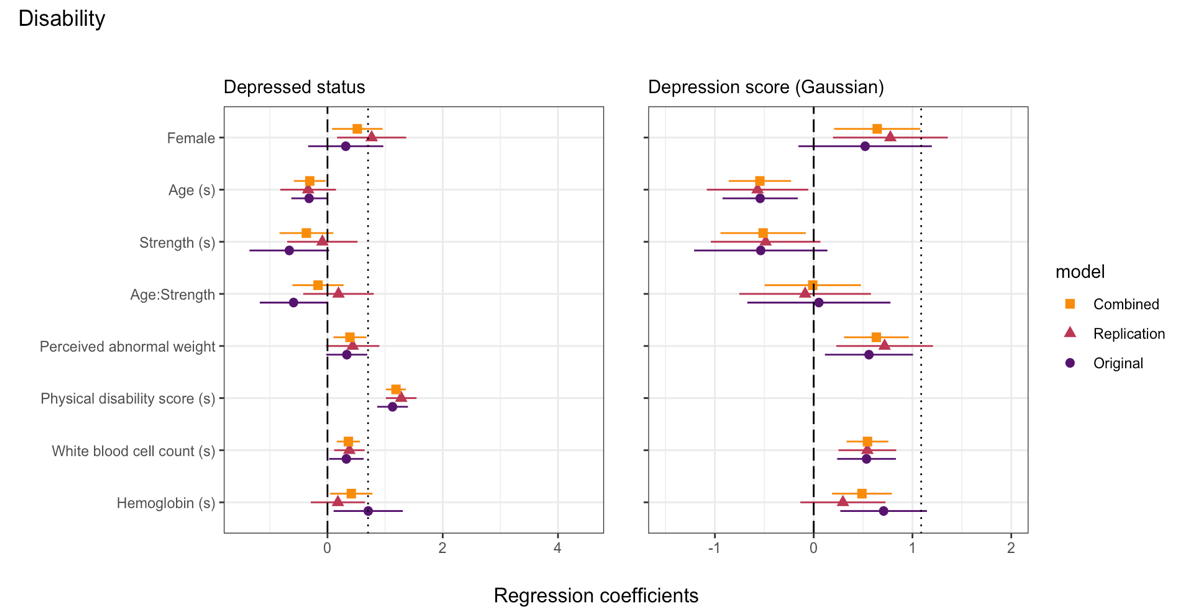


Figure S1: Coefficient plot for the Disability model of Depressed Status and Depression Score. Variables with (s) have been centered at their means and standardized by 2 SD. Dotted line marks the coefficient of sex alone.

## Questionable effort

For each grip strength test on each hand, NHANES technicians rated whether the participant exerted a maximal or questionable effort during the test.


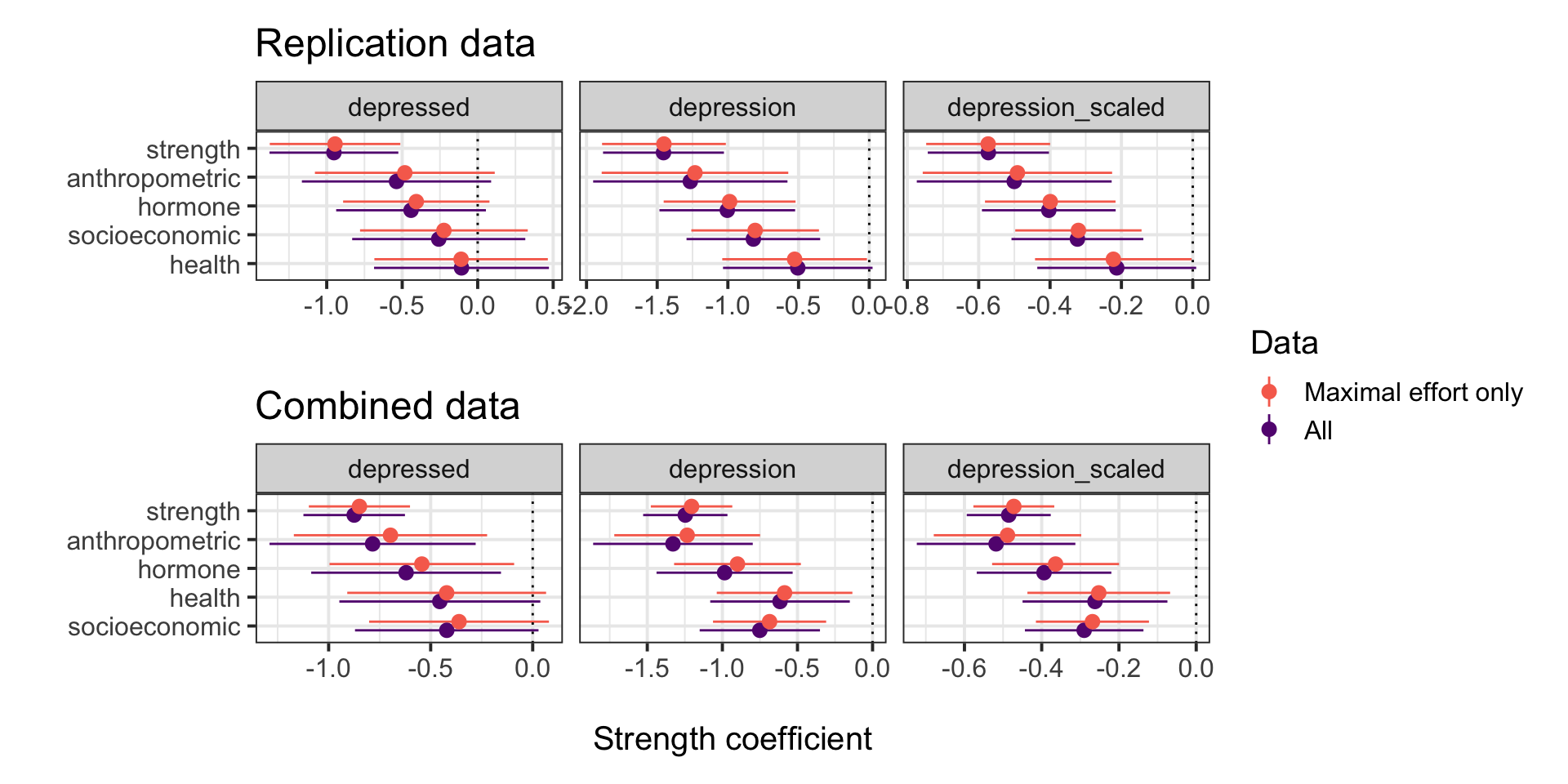


Figure S2: The strength regression coefficient for each model fit on Replication and Combined data with and without the questionable effort cases.

## Sex and strength coefficients in each model

Models fit on the combined data provide the most accurate estimates of each coefficient. Figure S3A shows the sex coefficient alone and in each model, all fit on the combined dataset. The magnitude of the coefficient for sex in each model is reduced compared to its effect alone, and is most reduced in the anthropometric model. Likewise, Figure S3B shows the magnitude of the strength coefficient in each model fit on the combined data. The coefficients are negative in all models (i.e., strength is protective), as predicted, but not statistically significant in the socioeconomic and health models of Depressed status, contrary to predictions. Figure S3C depicts the strength:age coefficients in each model.


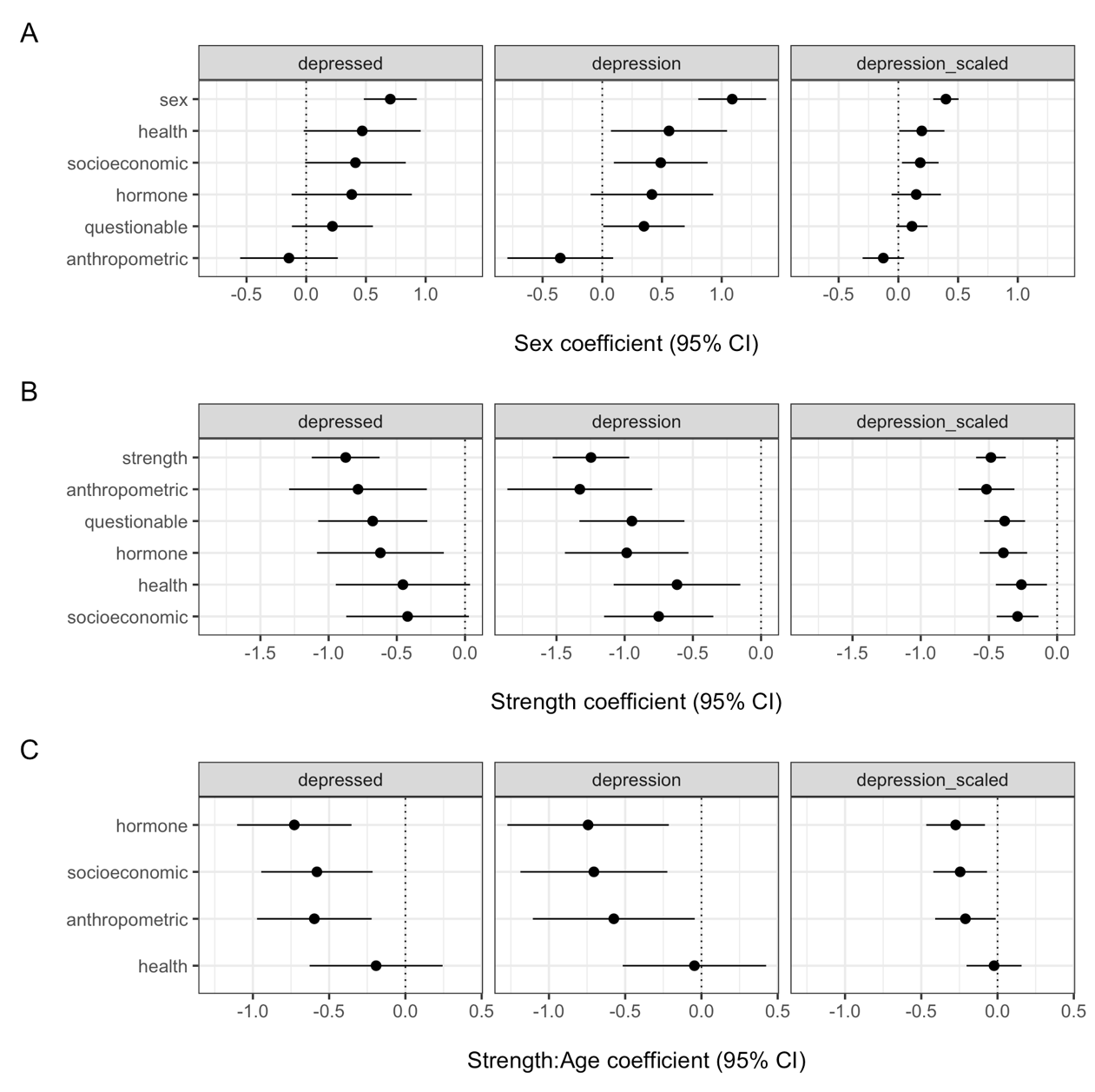


Figure S3: The regression coefficient for sex (A), strength (B) and the strength:age interaction (C) in each model. The ‘sex’ and ‘strength’ models include only sex and strength, respectively. Values are from models fit on the Combined dataset.

## Quasi-binomial models of depression score


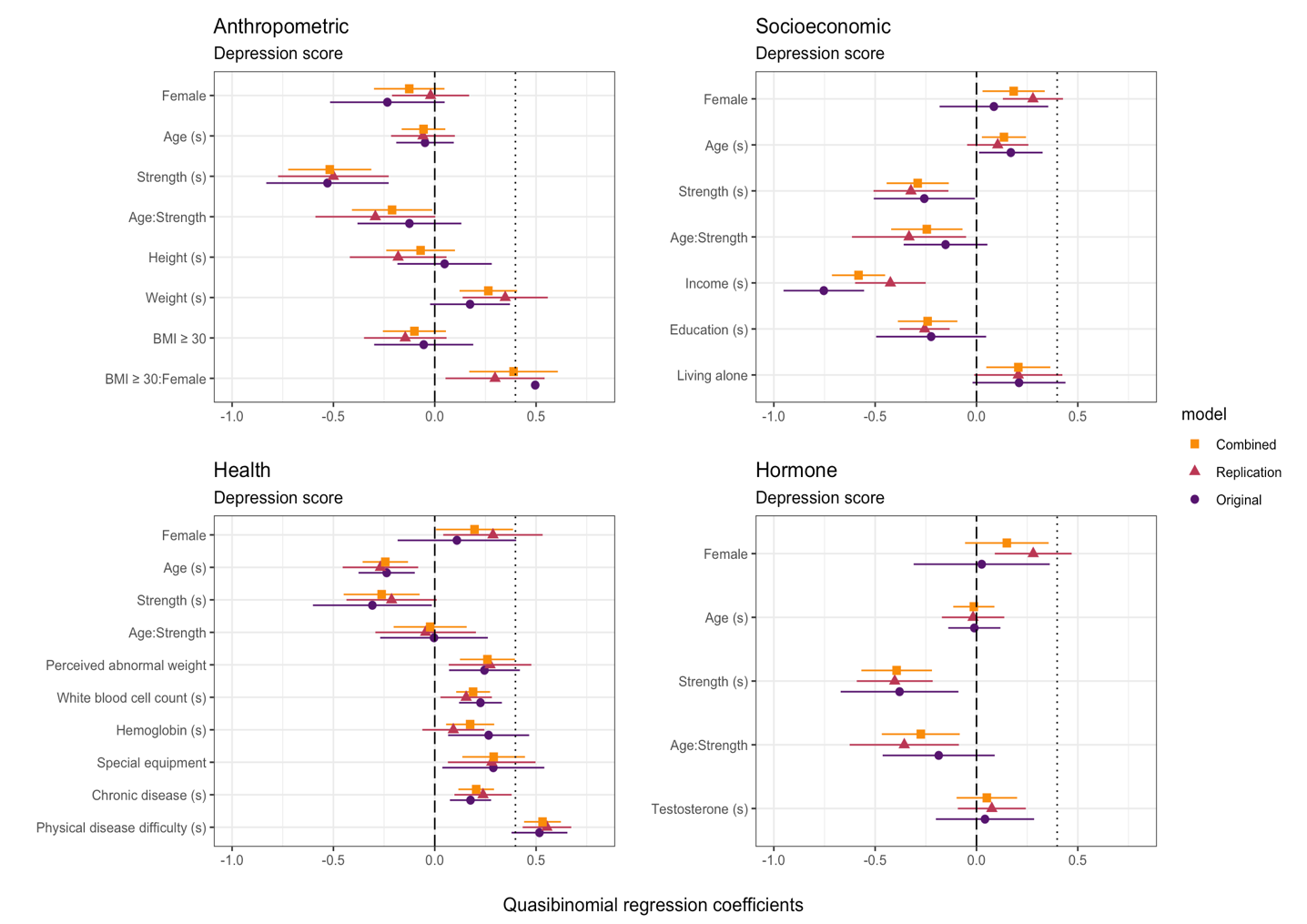


Figure S4: Coefficient plots for Anthropometric, Socioeconomic, Health, and Hormone quasibinomial models of Depression Score. Variables with (s) have been centered at their means and standardized by 2 SD. Dotted line marks the coefficient of sex alone.

## Regression tables

| Table S1: Regression coefficients of anthropometric models of depressed status (quasi-binomial). | | | | | | |
| --- | --- | --- | --- | --- | --- | --- |
| **Model** | **N** | **term** | **estimate** | **std.error** | **statistic** | **p.value** |
| Original | 3235 | (Intercept) | -2.44 | 0.23 | -10.63 | 0.00 |
|  |  | age_centered | 0.02 | 0.15 | 0.15 | 0.88 |
|  |  | strength_centered | -1.01 | 0.38 | -2.64 | 0.03 |
|  |  | sexfemale | -0.56 | 0.26 | -2.20 | 0.05 |
|  |  | BMI_category[30,85) | -0.02 | 0.28 | -0.08 | 0.94 |
|  |  | weight_centered | -0.08 | 0.23 | -0.36 | 0.73 |
|  |  | height_centered | 0.21 | 0.32 | 0.64 | 0.54 |
|  |  | age_centered:strength_centered | -0.75 | 0.24 | -3.07 | 0.01 |
|  |  | sexfemale:BMI_category[30,85) | 1.00 | 0.33 | 3.01 | 0.01 |
| Replication | 3650 | (Intercept) | -2.77 | 0.16 | -17.50 | 0.00 |
|  |  | age_centered | 0.01 | 0.18 | 0.04 | 0.97 |
|  |  | strength_centered | -0.54 | 0.32 | -1.68 | 0.14 |
|  |  | sexfemale | 0.32 | 0.30 | 1.06 | 0.32 |
|  |  | BMI_category[30,85) | 0.15 | 0.26 | 0.60 | 0.57 |
|  |  | weight_centered | 0.52 | 0.25 | 2.09 | 0.07 |
|  |  | height_centered | -0.50 | 0.29 | -1.69 | 0.13 |
|  |  | age_centered:strength_centered | -0.44 | 0.28 | -1.60 | 0.15 |
|  |  | sexfemale:BMI_category[30,85) | -0.05 | 0.32 | -0.16 | 0.88 |
| Combined | 6885 | (Intercept) | -2.56 | 0.15 | -17.15 | 0.00 |
|  |  | age_centered | -0.01 | 0.12 | -0.06 | 0.95 |
|  |  | strength_centered | -0.78 | 0.26 | -3.04 | 0.01 |
|  |  | sexfemale | -0.14 | 0.21 | -0.69 | 0.50 |
|  |  | BMI_category[30,85) | 0.02 | 0.19 | 0.10 | 0.92 |
|  |  | weight_centered | 0.26 | 0.18 | 1.49 | 0.15 |
|  |  | height_centered | -0.17 | 0.23 | -0.75 | 0.46 |
|  |  | age_centered:strength_centered | -0.60 | 0.19 | -3.12 | 0.00 |
|  |  | sexfemale:BMI_category[30,85) | 0.47 | 0.24 | 1.97 | 0.06 |

| Table S2: Regression coefficients of anthropometric models of depression score (Gaussian). | | | | | | |
| --- | --- | --- | --- | --- | --- | --- |
| **Model** | **N** | **term** | **estimate** | **std.error** | **statistic** | **p.value** |
| Original | 3235 | (Intercept) | 3.12 | 0.31 | 10.15 | 0.00 |
|  |  | age_centered | -0.11 | 0.20 | -0.56 | 0.59 |
|  |  | strength_centered | -1.38 | 0.41 | -3.36 | 0.01 |
|  |  | sexfemale | -0.63 | 0.37 | -1.69 | 0.13 |
|  |  | BMI_category[30,85) | -0.18 | 0.32 | -0.56 | 0.59 |
|  |  | weight_centered | 0.52 | 0.30 | 1.73 | 0.12 |
|  |  | height_centered | 0.11 | 0.32 | 0.34 | 0.74 |
|  |  | age_centered:strength_centered | -0.35 | 0.33 | -1.07 | 0.31 |
|  |  | sexfemale:BMI_category[30,85) | 1.55 | 0.52 | 2.99 | 0.02 |
| Replication | 3650 | (Intercept) | 3.04 | 0.14 | 21.99 | 0.00 |
|  |  | age_centered | -0.08 | 0.23 | -0.35 | 0.74 |
|  |  | strength_centered | -1.27 | 0.35 | -3.61 | 0.01 |
|  |  | sexfemale | -0.08 | 0.26 | -0.32 | 0.76 |
|  |  | BMI_category[30,85) | -0.48 | 0.26 | -1.88 | 0.10 |
|  |  | weight_centered | 1.05 | 0.33 | 3.14 | 0.02 |
|  |  | height_centered | -0.53 | 0.32 | -1.67 | 0.14 |
|  |  | age_centered:strength_centered | -0.80 | 0.43 | -1.86 | 0.10 |
|  |  | sexfemale:BMI_category[30,85) | 1.04 | 0.33 | 3.17 | 0.02 |
| Combined | 6885 | (Intercept) | 3.08 | 0.17 | 18.59 | 0.00 |
|  |  | age_centered | -0.11 | 0.15 | -0.69 | 0.50 |
|  |  | strength_centered | -1.33 | 0.27 | -4.90 | 0.00 |
|  |  | sexfemale | -0.35 | 0.23 | -1.56 | 0.13 |
|  |  | BMI_category[30,85) | -0.33 | 0.20 | -1.63 | 0.12 |
|  |  | weight_centered | 0.79 | 0.22 | 3.53 | 0.00 |
|  |  | height_centered | -0.22 | 0.23 | -0.96 | 0.35 |
|  |  | age_centered:strength_centered | -0.57 | 0.27 | -2.12 | 0.04 |
|  |  | sexfemale:BMI_category[30,85) | 1.28 | 0.30 | 4.28 | 0.00 |

| Table S3: Regression coefficients of anthropometric models of depression score (quasi-binomial). | | | | | | |
| --- | --- | --- | --- | --- | --- | --- |
| **Model** | **N** | **term** | **estimate** | **std.error** | **statistic** | **p.value** |
| Original | 3235 | (Intercept) | -2.05 | 0.11 | -17.90 | 0.00 |
|  |  | age_centered | -0.05 | 0.07 | -0.66 | 0.52 |
|  |  | strength_centered | -0.53 | 0.15 | -3.44 | 0.01 |
|  |  | sexfemale | -0.23 | 0.14 | -1.62 | 0.14 |
|  |  | BMI_category[30,85) | -0.05 | 0.12 | -0.44 | 0.67 |
|  |  | weight_centered | 0.17 | 0.10 | 1.73 | 0.12 |
|  |  | height_centered | 0.05 | 0.12 | 0.41 | 0.69 |
|  |  | age_centered:strength_centered | -0.12 | 0.13 | -0.96 | 0.36 |
|  |  | sexfemale:BMI_category[30,85) | 0.50 | 0.19 | 2.65 | 0.03 |
| Replication | 3650 | (Intercept) | -2.11 | 0.06 | -37.98 | 0.00 |
|  |  | age_centered | -0.06 | 0.08 | -0.72 | 0.49 |
|  |  | strength_centered | -0.50 | 0.14 | -3.59 | 0.01 |
|  |  | sexfemale | -0.02 | 0.10 | -0.21 | 0.84 |
|  |  | BMI_category[30,85) | -0.15 | 0.10 | -1.40 | 0.20 |
|  |  | weight_centered | 0.35 | 0.11 | 3.24 | 0.01 |
|  |  | height_centered | -0.18 | 0.12 | -1.48 | 0.18 |
|  |  | age_centered:strength_centered | -0.29 | 0.15 | -1.94 | 0.09 |
|  |  | sexfemale:BMI_category[30,85) | 0.30 | 0.12 | 2.38 | 0.05 |
| Combined | 6885 | (Intercept) | -2.08 | 0.06 | -32.29 | 0.00 |
|  |  | age_centered | -0.06 | 0.05 | -1.01 | 0.32 |
|  |  | strength_centered | -0.52 | 0.10 | -4.95 | 0.00 |
|  |  | sexfemale | -0.13 | 0.09 | -1.42 | 0.17 |
|  |  | BMI_category[30,85) | -0.10 | 0.08 | -1.26 | 0.22 |
|  |  | weight_centered | 0.26 | 0.07 | 3.64 | 0.00 |
|  |  | height_centered | -0.07 | 0.09 | -0.81 | 0.43 |
|  |  | age_centered:strength_centered | -0.21 | 0.10 | -2.08 | 0.05 |
|  |  | sexfemale:BMI_category[30,85) | 0.39 | 0.11 | 3.49 | 0.00 |

| Table S4: Regression coefficients of socioeconomic models of depressed status (quasi-binomial). | | | | | | |
| --- | --- | --- | --- | --- | --- | --- |
| **Model** | **N** | **term** | **estimate** | **std.error** | **statistic** | **p.value** |
| Original | 3033 | (Intercept) | -2.73 | 0.26 | -10.50 | 0.00 |
|  |  | age_centered | 0.37 | 0.24 | 1.55 | 0.15 |
|  |  | strength_centered | -0.58 | 0.35 | -1.68 | 0.12 |
|  |  | sexfemale | 0.07 | 0.35 | 0.20 | 0.85 |
|  |  | income_centered | -1.27 | 0.28 | -4.50 | 0.00 |
|  |  | edu_centered | -0.52 | 0.32 | -1.64 | 0.13 |
|  |  | living_alone | 0.31 | 0.32 | 0.98 | 0.35 |
|  |  | age_centered:strength_centered | -0.76 | 0.24 | -3.11 | 0.01 |
| Replication | 3403 | (Intercept) | -3.07 | 0.18 | -16.80 | 0.00 |
|  |  | age_centered | 0.31 | 0.18 | 1.70 | 0.13 |
|  |  | strength_centered | -0.26 | 0.29 | -0.88 | 0.40 |
|  |  | sexfemale | 0.77 | 0.23 | 3.38 | 0.01 |
|  |  | income_centered | -0.83 | 0.31 | -2.66 | 0.03 |
|  |  | edu_centered | -0.55 | 0.22 | -2.46 | 0.04 |
|  |  | living_alone | 0.48 | 0.22 | 2.22 | 0.06 |
|  |  | age_centered:strength_centered | -0.40 | 0.26 | -1.52 | 0.17 |
| Combined | 6436 | (Intercept) | -2.88 | 0.16 | -17.55 | 0.00 |
|  |  | age_centered | 0.32 | 0.14 | 2.25 | 0.03 |
|  |  | strength_centered | -0.42 | 0.23 | -1.84 | 0.08 |
|  |  | sexfemale | 0.41 | 0.22 | 1.92 | 0.07 |
|  |  | income_centered | -1.03 | 0.21 | -4.90 | 0.00 |
|  |  | edu_centered | -0.54 | 0.19 | -2.78 | 0.01 |
|  |  | living_alone | 0.40 | 0.19 | 2.06 | 0.05 |
|  |  | age_centered:strength_centered | -0.58 | 0.19 | -3.12 | 0.00 |

| Table S5: Regression coefficients of socioeconomic models of depression score (Gaussian). | | | | | | |
| --- | --- | --- | --- | --- | --- | --- |
| **Model** | **N** | **term** | **estimate** | **std.error** | **statistic** | **p.value** |
| Original | 3033 | (Intercept) | 2.91 | 0.24 | 12.31 | 0.00 |
|  |  | age_centered | 0.48 | 0.22 | 2.22 | 0.05 |
|  |  | strength_centered | -0.68 | 0.34 | -2.02 | 0.07 |
|  |  | sexfemale | 0.23 | 0.36 | 0.64 | 0.54 |
|  |  | income_centered | -1.95 | 0.30 | -6.58 | 0.00 |
|  |  | edu_centered | -0.65 | 0.42 | -1.55 | 0.15 |
|  |  | living_alone | 0.64 | 0.34 | 1.87 | 0.09 |
|  |  | age_centered:strength_centered | -0.47 | 0.27 | -1.72 | 0.12 |
| Replication | 3403 | (Intercept) | 2.64 | 0.12 | 21.48 | 0.00 |
|  |  | age_centered | 0.40 | 0.22 | 1.86 | 0.10 |
|  |  | strength_centered | -0.82 | 0.24 | -3.40 | 0.01 |
|  |  | sexfemale | 0.74 | 0.19 | 3.99 | 0.00 |
|  |  | income_centered | -1.13 | 0.23 | -4.97 | 0.00 |
|  |  | edu_centered | -0.72 | 0.18 | -3.99 | 0.00 |
|  |  | living_alone | 0.57 | 0.33 | 1.74 | 0.12 |
|  |  | age_centered:strength_centered | -0.94 | 0.41 | -2.26 | 0.05 |
| Combined | 6436 | (Intercept) | 2.77 | 0.13 | 20.70 | 0.00 |
|  |  | age_centered | 0.44 | 0.15 | 2.83 | 0.01 |
|  |  | strength_centered | -0.75 | 0.20 | -3.67 | 0.00 |
|  |  | sexfemale | 0.49 | 0.20 | 2.44 | 0.02 |
|  |  | income_centered | -1.53 | 0.18 | -8.38 | 0.00 |
|  |  | edu_centered | -0.69 | 0.22 | -3.12 | 0.00 |
|  |  | living_alone | 0.60 | 0.24 | 2.54 | 0.02 |
|  |  | age_centered:strength_centered | -0.71 | 0.25 | -2.87 | 0.01 |

| Table S6: Regression coefficients of socioeconomic models of depression score (quasi-binomial). | | | | | | |
| --- | --- | --- | --- | --- | --- | --- |
| **Model** | **N** | **term** | **estimate** | **std.error** | **statistic** | **p.value** |
| Original | 3033 | (Intercept) | -2.19 | 0.09 | -23.17 | 0.00 |
|  |  | age_centered | 0.17 | 0.08 | 2.12 | 0.06 |
|  |  | strength_centered | -0.26 | 0.13 | -2.02 | 0.07 |
|  |  | sexfemale | 0.09 | 0.14 | 0.63 | 0.54 |
|  |  | income_centered | -0.75 | 0.10 | -7.43 | 0.00 |
|  |  | edu_centered | -0.22 | 0.14 | -1.62 | 0.14 |
|  |  | living_alone | 0.21 | 0.12 | 1.79 | 0.10 |
|  |  | age_centered:strength_centered | -0.15 | 0.11 | -1.45 | 0.18 |
| Replication | 3403 | (Intercept) | -2.28 | 0.06 | -40.50 | 0.00 |
|  |  | age_centered | 0.10 | 0.08 | 1.36 | 0.21 |
|  |  | strength_centered | -0.32 | 0.09 | -3.43 | 0.01 |
|  |  | sexfemale | 0.28 | 0.08 | 3.68 | 0.01 |
|  |  | income_centered | -0.42 | 0.09 | -4.78 | 0.00 |
|  |  | edu_centered | -0.26 | 0.06 | -4.06 | 0.00 |
|  |  | living_alone | 0.21 | 0.11 | 1.86 | 0.10 |
|  |  | age_centered:strength_centered | -0.33 | 0.14 | -2.32 | 0.05 |
| Combined | 6436 | (Intercept) | -2.23 | 0.06 | -39.69 | 0.00 |
|  |  | age_centered | 0.14 | 0.06 | 2.44 | 0.02 |
|  |  | strength_centered | -0.29 | 0.08 | -3.70 | 0.00 |
|  |  | sexfemale | 0.18 | 0.08 | 2.34 | 0.03 |
|  |  | income_centered | -0.58 | 0.07 | -8.68 | 0.00 |
|  |  | edu_centered | -0.24 | 0.08 | -3.21 | 0.00 |
|  |  | living_alone | 0.21 | 0.08 | 2.56 | 0.02 |
|  |  | age_centered:strength_centered | -0.25 | 0.09 | -2.73 | 0.01 |

| Table S7: Regression coefficients of health models of depressed status (quasi-binomial). | | | | | | |
| --- | --- | --- | --- | --- | --- | --- |
| **Model** | **N** | **term** | **estimate** | **std.error** | **statistic** | **p.value** |
| Original | 2866 | (Intercept) | -3.01 | 0.25 | -12.08 | 0.00 |
|  |  | age_centered | -0.36 | 0.17 | -2.13 | 0.07 |
|  |  | strength_centered | -0.81 | 0.38 | -2.13 | 0.07 |
|  |  | sexfemale | 0.20 | 0.39 | 0.52 | 0.62 |
|  |  | perceived_abnormal_weightTRUE | 0.35 | 0.18 | 1.95 | 0.09 |
|  |  | physical_disease_count_centered | 0.67 | 0.15 | 4.36 | 0.00 |
|  |  | chronic_disease_score_centered | 0.35 | 0.16 | 2.24 | 0.06 |
|  |  | special_equipmentTRUE | 0.62 | 0.22 | 2.77 | 0.03 |
|  |  | whitebloodcell_centered | 0.36 | 0.14 | 2.49 | 0.04 |
|  |  | hemoglobin_centered | 0.75 | 0.33 | 2.26 | 0.06 |
|  |  | age_centered:strength_centered | -0.60 | 0.28 | -2.13 | 0.07 |
| Replication | 3260 | (Intercept) | -3.34 | 0.18 | -18.92 | 0.00 |
|  |  | age_centered | -0.38 | 0.22 | -1.74 | 0.14 |
|  |  | strength_centered | -0.11 | 0.30 | -0.36 | 0.73 |
|  |  | sexfemale | 0.78 | 0.31 | 2.53 | 0.05 |
|  |  | perceived_abnormal_weightTRUE | 0.38 | 0.25 | 1.54 | 0.18 |
|  |  | physical_disease_count_centered | 0.89 | 0.08 | 10.49 | 0.00 |
|  |  | chronic_disease_score_centered | 0.42 | 0.17 | 2.45 | 0.06 |
|  |  | special_equipmentTRUE | 0.42 | 0.33 | 1.29 | 0.25 |
|  |  | whitebloodcell_centered | 0.30 | 0.16 | 1.84 | 0.13 |
|  |  | hemoglobin_centered | 0.17 | 0.25 | 0.70 | 0.51 |
|  |  | age_centered:strength_centered | 0.17 | 0.34 | 0.52 | 0.63 |
| Combined | 6126 | (Intercept) | -3.15 | 0.16 | -20.06 | 0.00 |
|  |  | age_centered | -0.35 | 0.13 | -2.63 | 0.02 |
|  |  | strength_centered | -0.46 | 0.25 | -1.81 | 0.08 |
|  |  | sexfemale | 0.47 | 0.25 | 1.88 | 0.07 |
|  |  | perceived_abnormal_weightTRUE | 0.37 | 0.15 | 2.49 | 0.02 |
|  |  | physical_disease_count_centered | 0.77 | 0.08 | 10.00 | 0.00 |
|  |  | chronic_disease_score_centered | 0.37 | 0.12 | 3.14 | 0.00 |
|  |  | special_equipmentTRUE | 0.50 | 0.19 | 2.67 | 0.01 |
|  |  | whitebloodcell_centered | 0.33 | 0.11 | 3.05 | 0.01 |
|  |  | hemoglobin_centered | 0.44 | 0.19 | 2.25 | 0.03 |
|  |  | age_centered:strength_centered | -0.19 | 0.22 | -0.86 | 0.40 |

| Table S8: Regression coefficients of health models of depression score. (Gaussian) | | | | | | |
| --- | --- | --- | --- | --- | --- | --- |
| **Model** | **N** | **term** | **estimate** | **std.error** | **statistic** | **p.value** |
| Original | 2866 | (Intercept) | 2.45 | 0.24 | 10.42 | 0.00 |
|  |  | age_centered | -0.63 | 0.19 | -3.31 | 0.01 |
|  |  | strength_centered | -0.72 | 0.39 | -1.83 | 0.11 |
|  |  | sexfemale | 0.35 | 0.40 | 0.88 | 0.41 |
|  |  | perceived_abnormal_weightTRUE | 0.61 | 0.21 | 2.86 | 0.02 |
|  |  | physical_disease_count_centered | 2.15 | 0.34 | 6.40 | 0.00 |
|  |  | chronic_disease_score_centered | 0.50 | 0.16 | 3.11 | 0.02 |
|  |  | special_equipmentTRUE | 1.37 | 0.57 | 2.40 | 0.05 |
|  |  | whitebloodcell_centered | 0.62 | 0.16 | 3.87 | 0.01 |
|  |  | hemoglobin_centered | 0.70 | 0.25 | 2.77 | 0.03 |
|  |  | age_centered:strength_centered | -0.02 | 0.35 | -0.04 | 0.97 |
| Replication | 3260 | (Intercept) | 2.24 | 0.20 | 11.19 | 0.00 |
|  |  | age_centered | -0.68 | 0.24 | -2.89 | 0.03 |
|  |  | strength_centered | -0.51 | 0.27 | -1.87 | 0.12 |
|  |  | sexfemale | 0.77 | 0.30 | 2.53 | 0.05 |
|  |  | perceived_abnormal_weightTRUE | 0.64 | 0.25 | 2.57 | 0.05 |
|  |  | physical_disease_count_centered | 2.31 | 0.32 | 7.22 | 0.00 |
|  |  | chronic_disease_score_centered | 0.74 | 0.20 | 3.71 | 0.01 |
|  |  | special_equipmentTRUE | 1.30 | 0.48 | 2.73 | 0.04 |
|  |  | whitebloodcell_centered | 0.45 | 0.18 | 2.49 | 0.05 |
|  |  | hemoglobin_centered | 0.26 | 0.21 | 1.22 | 0.28 |
|  |  | age_centered:strength_centered | -0.07 | 0.33 | -0.21 | 0.84 |
| Combined | 6126 | (Intercept) | 2.35 | 0.15 | 15.23 | 0.00 |
|  |  | age_centered | -0.64 | 0.15 | -4.36 | 0.00 |
|  |  | strength_centered | -0.62 | 0.24 | -2.60 | 0.02 |
|  |  | sexfemale | 0.56 | 0.25 | 2.25 | 0.03 |
|  |  | perceived_abnormal_weightTRUE | 0.63 | 0.17 | 3.78 | 0.00 |
|  |  | physical_disease_count_centered | 2.23 | 0.24 | 9.48 | 0.00 |
|  |  | chronic_disease_score_centered | 0.62 | 0.13 | 4.78 | 0.00 |
|  |  | special_equipmentTRUE | 1.34 | 0.35 | 3.86 | 0.00 |
|  |  | whitebloodcell_centered | 0.53 | 0.12 | 4.41 | 0.00 |
|  |  | hemoglobin_centered | 0.47 | 0.16 | 2.94 | 0.01 |
|  |  | age_centered:strength_centered | -0.05 | 0.24 | -0.19 | 0.85 |

| Table S9: Regression coefficients of health models of depression score. (quasi-binomial) | | | | | | |
| --- | --- | --- | --- | --- | --- | --- |
| **Model** | **N** | **term** | **estimate** | **std.error** | **statistic** | **p.value** |
| Original | 2866 | (Intercept) | -2.35 | 0.10 | -23.07 | 0.00 |
|  |  | age_centered | -0.24 | 0.07 | -3.35 | 0.01 |
|  |  | strength_centered | -0.31 | 0.15 | -2.06 | 0.08 |
|  |  | sexfemale | 0.11 | 0.15 | 0.73 | 0.49 |
|  |  | perceived_abnormal_weightTRUE | 0.25 | 0.09 | 2.75 | 0.03 |
|  |  | physical_disease_count_centered | 0.52 | 0.07 | 7.34 | 0.00 |
|  |  | chronic_disease_score_centered | 0.18 | 0.05 | 3.41 | 0.01 |
|  |  | special_equipmentTRUE | 0.29 | 0.13 | 2.25 | 0.06 |
|  |  | whitebloodcell_centered | 0.23 | 0.05 | 4.18 | 0.00 |
|  |  | hemoglobin_centered | 0.27 | 0.10 | 2.61 | 0.04 |
|  |  | age_centered:strength_centered | 0.00 | 0.14 | -0.03 | 0.98 |
| Replication | 3260 | (Intercept) | -2.47 | 0.09 | -28.89 | 0.00 |
|  |  | age_centered | -0.27 | 0.10 | -2.82 | 0.04 |
|  |  | strength_centered | -0.21 | 0.11 | -1.88 | 0.12 |
|  |  | sexfemale | 0.29 | 0.13 | 2.29 | 0.07 |
|  |  | perceived_abnormal_weightTRUE | 0.27 | 0.10 | 2.62 | 0.05 |
|  |  | physical_disease_count_centered | 0.55 | 0.06 | 9.04 | 0.00 |
|  |  | chronic_disease_score_centered | 0.24 | 0.07 | 3.31 | 0.02 |
|  |  | special_equipmentTRUE | 0.28 | 0.11 | 2.55 | 0.05 |
|  |  | whitebloodcell_centered | 0.16 | 0.06 | 2.39 | 0.06 |
|  |  | hemoglobin_centered | 0.09 | 0.08 | 1.18 | 0.29 |
|  |  | age_centered:strength_centered | -0.05 | 0.13 | -0.36 | 0.74 |
| Combined | 6126 | (Intercept) | -2.41 | 0.07 | -36.11 | 0.00 |
|  |  | age_centered | -0.24 | 0.06 | -4.27 | 0.00 |
|  |  | strength_centered | -0.26 | 0.10 | -2.74 | 0.01 |
|  |  | sexfemale | 0.20 | 0.10 | 2.02 | 0.06 |
|  |  | perceived_abnormal_weightTRUE | 0.26 | 0.07 | 3.75 | 0.00 |
|  |  | physical_disease_count_centered | 0.53 | 0.05 | 11.45 | 0.00 |
|  |  | chronic_disease_score_centered | 0.20 | 0.04 | 4.55 | 0.00 |
|  |  | special_equipmentTRUE | 0.29 | 0.08 | 3.68 | 0.00 |
|  |  | whitebloodcell_centered | 0.19 | 0.04 | 4.44 | 0.00 |
|  |  | hemoglobin_centered | 0.17 | 0.06 | 2.88 | 0.01 |
|  |  | age_centered:strength_centered | -0.02 | 0.09 | -0.25 | 0.81 |

| Table S10: Regression coefficients of hormone models of depressed status (quasi-binomial). | | | | | | |
| --- | --- | --- | --- | --- | --- | --- |
| **Model** | **N** | **term** | **estimate** | **std.error** | **statistic** | **p.value** |
| Original | 3033 | (Intercept) | -2.55 | 0.24 | -10.49 | 0.00 |
|  |  | age_centered | 0.01 | 0.16 | 0.08 | 0.93 |
|  |  | strength_centered | -0.78 | 0.38 | -2.05 | 0.06 |
|  |  | sexfemale | 0.05 | 0.38 | 0.13 | 0.90 |
|  |  | testosterone_centered | 0.12 | 0.34 | 0.36 | 0.72 |
|  |  | age_centered:strength_centered | -0.96 | 0.27 | -3.59 | 0.00 |
| Replication | 3509 | (Intercept) | -2.90 | 0.17 | -17.21 | 0.00 |
|  |  | age_centered | 0.07 | 0.18 | 0.38 | 0.72 |
|  |  | strength_centered | -0.44 | 0.25 | -1.74 | 0.11 |
|  |  | sexfemale | 0.76 | 0.27 | 2.82 | 0.02 |
|  |  | testosterone_centered | 0.19 | 0.25 | 0.78 | 0.45 |
|  |  | age_centered:strength_centered | -0.52 | 0.26 | -1.99 | 0.08 |
| Combined | 6542 | (Intercept) | -2.71 | 0.16 | -16.62 | 0.00 |
|  |  | age_centered | 0.03 | 0.12 | 0.28 | 0.78 |
|  |  | strength_centered | -0.62 | 0.24 | -2.62 | 0.01 |
|  |  | sexfemale | 0.38 | 0.26 | 1.48 | 0.15 |
|  |  | testosterone_centered | 0.13 | 0.22 | 0.62 | 0.54 |
|  |  | age_centered:strength_centered | -0.73 | 0.19 | -3.81 | 0.00 |

| Table S11: Regression coefficients of hormone models of depression score. (Gaussian) | | | | | | |
| --- | --- | --- | --- | --- | --- | --- |
| **Model** | **N** | **term** | **estimate** | **std.error** | **statistic** | **p.value** |
| Original | 3033 | (Intercept) | 2.96 | 0.29 | 10.08 | 0.00 |
|  |  | age_centered | 0.00 | 0.18 | 0.03 | 0.98 |
|  |  | strength_centered | -0.96 | 0.39 | -2.45 | 0.03 |
|  |  | sexfemale | 0.08 | 0.44 | 0.18 | 0.86 |
|  |  | testosterone_centered | 0.10 | 0.30 | 0.32 | 0.76 |
|  |  | age_centered:strength_centered | -0.50 | 0.36 | -1.38 | 0.19 |
| Replication | 3509 | (Intercept) | 2.66 | 0.16 | 16.29 | 0.00 |
|  |  | age_centered | 0.05 | 0.22 | 0.23 | 0.82 |
|  |  | strength_centered | -1.00 | 0.24 | -4.10 | 0.00 |
|  |  | sexfemale | 0.75 | 0.23 | 3.30 | 0.01 |
|  |  | testosterone_centered | 0.16 | 0.19 | 0.82 | 0.43 |
|  |  | age_centered:strength_centered | -0.97 | 0.41 | -2.40 | 0.04 |
| Combined | 6542 | (Intercept) | 2.81 | 0.17 | 16.13 | 0.00 |
|  |  | age_centered | 0.03 | 0.15 | 0.19 | 0.85 |
|  |  | strength_centered | -0.99 | 0.23 | -4.26 | 0.00 |
|  |  | sexfemale | 0.42 | 0.26 | 1.58 | 0.12 |
|  |  | testosterone_centered | 0.11 | 0.18 | 0.63 | 0.53 |
|  |  | age_centered:strength_centered | -0.74 | 0.27 | -2.75 | 0.01 |

| Table S12: Regression coefficients of hormone models of depression score. (quasi-binomial) | | | | | | |
| --- | --- | --- | --- | --- | --- | --- |
| **Model** | **N** | **term** | **estimate** | **std.error** | **statistic** | **p.value** |
| Original | 3033 | (Intercept) | -2.11 | 0.11 | -18.87 | 0.00 |
|  |  | age_centered | -0.01 | 0.07 | -0.16 | 0.88 |
|  |  | strength_centered | -0.38 | 0.15 | -2.56 | 0.03 |
|  |  | sexfemale | 0.03 | 0.17 | 0.15 | 0.88 |
|  |  | testosterone_centered | 0.04 | 0.12 | 0.34 | 0.74 |
|  |  | age_centered:strength_centered | -0.19 | 0.14 | -1.32 | 0.21 |
| Replication | 3509 | (Intercept) | -2.24 | 0.07 | -32.70 | 0.00 |
|  |  | age_centered | -0.02 | 0.08 | -0.21 | 0.83 |
|  |  | strength_centered | -0.40 | 0.10 | -4.22 | 0.00 |
|  |  | sexfemale | 0.28 | 0.10 | 2.89 | 0.02 |
|  |  | testosterone_centered | 0.08 | 0.09 | 0.88 | 0.40 |
|  |  | age_centered:strength_centered | -0.36 | 0.14 | -2.59 | 0.03 |
| Combined | 6542 | (Intercept) | -2.17 | 0.07 | -31.15 | 0.00 |
|  |  | age_centered | -0.01 | 0.05 | -0.25 | 0.80 |
|  |  | strength_centered | -0.39 | 0.09 | -4.43 | 0.00 |
|  |  | sexfemale | 0.15 | 0.11 | 1.42 | 0.17 |
|  |  | testosterone_centered | 0.05 | 0.08 | 0.67 | 0.51 |
|  |  | age_centered:strength_centered | -0.27 | 0.10 | -2.80 | 0.01 |

| Table S13: Regression coefficients of disability models of depressed status (quasi-binomial). | | | | | | |
| --- | --- | --- | --- | --- | --- | --- |
| **Model** | **N** | **term** | **estimate** | **std.error** | **statistic** | **p.value** |
| Original | 2879 | (Intercept) | -3.04 | 0.24 | -12.76 | 0.00 |
|  |  | age_centered | -0.32 | 0.16 | -2.02 | 0.07 |
|  |  | strength_centered | -0.66 | 0.35 | -1.88 | 0.09 |
|  |  | sexfemale | 0.32 | 0.33 | 0.95 | 0.37 |
|  |  | perceived_abnormal_weightTRUE | 0.34 | 0.18 | 1.85 | 0.10 |
|  |  | disability_score_centered | 1.13 | 0.14 | 8.29 | 0.00 |
|  |  | whitebloodcell_centered | 0.33 | 0.15 | 2.13 | 0.06 |
|  |  | hemoglobin_centered | 0.71 | 0.31 | 2.31 | 0.05 |
|  |  | age_centered:strength_centered | -0.59 | 0.30 | -1.97 | 0.08 |
| Replication | 3277 | (Intercept) | -3.33 | 0.19 | -17.74 | 0.00 |
|  |  | age_centered | -0.34 | 0.25 | -1.36 | 0.22 |
|  |  | strength_centered | -0.09 | 0.31 | -0.28 | 0.78 |
|  |  | sexfemale | 0.77 | 0.31 | 2.51 | 0.04 |
|  |  | perceived_abnormal_weightTRUE | 0.44 | 0.24 | 1.85 | 0.11 |
|  |  | disability_score_centered | 1.28 | 0.14 | 9.42 | 0.00 |
|  |  | whitebloodcell_centered | 0.38 | 0.14 | 2.80 | 0.03 |
|  |  | hemoglobin_centered | 0.18 | 0.24 | 0.75 | 0.48 |
|  |  | age_centered:strength_centered | 0.19 | 0.31 | 0.61 | 0.56 |
| Combined | 6156 | (Intercept) | -3.16 | 0.15 | -20.51 | 0.00 |
|  |  | age_centered | -0.31 | 0.14 | -2.19 | 0.04 |
|  |  | strength_centered | -0.37 | 0.24 | -1.54 | 0.14 |
|  |  | sexfemale | 0.52 | 0.22 | 2.31 | 0.03 |
|  |  | perceived_abnormal_weightTRUE | 0.39 | 0.15 | 2.67 | 0.01 |
|  |  | disability_score_centered | 1.19 | 0.09 | 13.42 | 0.00 |
|  |  | whitebloodcell_centered | 0.36 | 0.10 | 3.53 | 0.00 |
|  |  | hemoglobin_centered | 0.41 | 0.19 | 2.20 | 0.04 |
|  |  | age_centered:strength_centered | -0.16 | 0.23 | -0.72 | 0.48 |

| Table S14: Regression coefficients of disability models of depression score. (Gaussian) | | | | | | |
| --- | --- | --- | --- | --- | --- | --- |
| **Model** | **N** | **term** | **estimate** | **std.error** | **statistic** | **p.value** |
| Original | 2879 | (Intercept) | 2.50 | 0.22 | 11.32 | 0.00 |
|  |  | age_centered | -0.54 | 0.19 | -2.79 | 0.02 |
|  |  | strength_centered | -0.54 | 0.34 | -1.56 | 0.15 |
|  |  | sexfemale | 0.52 | 0.34 | 1.51 | 0.16 |
|  |  | perceived_abnormal_weightTRUE | 0.56 | 0.23 | 2.46 | 0.04 |
|  |  | disability_score_centered | 3.19 | 0.49 | 6.55 | 0.00 |
|  |  | whitebloodcell_centered | 0.54 | 0.15 | 3.52 | 0.01 |
|  |  | hemoglobin_centered | 0.71 | 0.22 | 3.16 | 0.01 |
|  |  | age_centered:strength_centered | 0.05 | 0.37 | 0.14 | 0.89 |
| Replication | 3277 | (Intercept) | 2.29 | 0.18 | 12.85 | 0.00 |
|  |  | age_centered | -0.57 | 0.26 | -2.17 | 0.07 |
|  |  | strength_centered | -0.49 | 0.28 | -1.72 | 0.13 |
|  |  | sexfemale | 0.78 | 0.30 | 2.62 | 0.03 |
|  |  | perceived_abnormal_weightTRUE | 0.72 | 0.25 | 2.87 | 0.02 |
|  |  | disability_score_centered | 3.05 | 0.29 | 10.62 | 0.00 |
|  |  | whitebloodcell_centered | 0.54 | 0.15 | 3.64 | 0.01 |
|  |  | hemoglobin_centered | 0.30 | 0.22 | 1.35 | 0.22 |
|  |  | age_centered:strength_centered | -0.09 | 0.34 | -0.25 | 0.81 |
| Combined | 6156 | (Intercept) | 2.40 | 0.14 | 16.81 | 0.00 |
|  |  | age_centered | -0.54 | 0.16 | -3.38 | 0.00 |
|  |  | strength_centered | -0.51 | 0.22 | -2.32 | 0.03 |
|  |  | sexfemale | 0.64 | 0.22 | 2.88 | 0.01 |
|  |  | perceived_abnormal_weightTRUE | 0.63 | 0.17 | 3.79 | 0.00 |
|  |  | disability_score_centered | 3.11 | 0.26 | 11.84 | 0.00 |
|  |  | whitebloodcell_centered | 0.54 | 0.11 | 5.05 | 0.00 |
|  |  | hemoglobin_centered | 0.49 | 0.15 | 3.16 | 0.00 |
|  |  | age_centered:strength_centered | -0.01 | 0.25 | -0.04 | 0.97 |

| Table S15: Regression coefficients of disability models of depression score. (quasi-binomial) | | | | | | |
| --- | --- | --- | --- | --- | --- | --- |
| **Model** | **N** | **term** | **estimate** | **std.error** | **statistic** | **p.value** |
| Original | 2879 | (Intercept) | -2.36 | 0.10 | -24.40 | 0.00 |
|  |  | age_centered | -0.20 | 0.07 | -2.68 | 0.03 |
|  |  | strength_centered | -0.24 | 0.13 | -1.89 | 0.09 |
|  |  | sexfemale | 0.17 | 0.12 | 1.39 | 0.20 |
|  |  | perceived_abnormal_weightTRUE | 0.23 | 0.09 | 2.47 | 0.04 |
|  |  | disability_score_centered | 0.76 | 0.09 | 8.51 | 0.00 |
|  |  | whitebloodcell_centered | 0.20 | 0.05 | 3.77 | 0.00 |
|  |  | hemoglobin_centered | 0.26 | 0.09 | 2.95 | 0.02 |
|  |  | age_centered:strength_centered | 0.01 | 0.15 | 0.09 | 0.93 |
| Replication | 3277 | (Intercept) | -2.46 | 0.08 | -30.14 | 0.00 |
|  |  | age_centered | -0.23 | 0.10 | -2.23 | 0.06 |
|  |  | strength_centered | -0.21 | 0.11 | -1.88 | 0.10 |
|  |  | sexfemale | 0.28 | 0.12 | 2.35 | 0.05 |
|  |  | perceived_abnormal_weightTRUE | 0.30 | 0.10 | 2.90 | 0.02 |
|  |  | disability_score_centered | 0.75 | 0.06 | 13.10 | 0.00 |
|  |  | whitebloodcell_centered | 0.20 | 0.05 | 3.72 | 0.01 |
|  |  | hemoglobin_centered | 0.10 | 0.07 | 1.37 | 0.21 |
|  |  | age_centered:strength_centered | -0.05 | 0.12 | -0.38 | 0.72 |
| Combined | 6156 | (Intercept) | -2.41 | 0.06 | -37.73 | 0.00 |
|  |  | age_centered | -0.21 | 0.06 | -3.33 | 0.00 |
|  |  | strength_centered | -0.23 | 0.09 | -2.65 | 0.01 |
|  |  | sexfemale | 0.22 | 0.08 | 2.61 | 0.02 |
|  |  | perceived_abnormal_weightTRUE | 0.26 | 0.07 | 3.79 | 0.00 |
|  |  | disability_score_centered | 0.75 | 0.05 | 15.37 | 0.00 |
|  |  | whitebloodcell_centered | 0.20 | 0.04 | 5.23 | 0.00 |
|  |  | hemoglobin_centered | 0.18 | 0.06 | 3.14 | 0.00 |
|  |  | age_centered:strength_centered | -0.01 | 0.09 | -0.12 | 0.91 |

## Change in the sex difference in depression from 2011-2012 to 2013-2014

To test if the sex difference in depression changes from 2011-2012 (G series) to 2013-2014 (H series), we fit regression models of depressed status and depression score with sex plus an interaction term with data series, but no other control variables.

| Table S16: This model tested an interaction of survey cycle and sex on depression. Shown here is a significant interaction such that in the Replication dataset female sex was a stronger risk factor for depression. | | | | | | |
| --- | --- | --- | --- | --- | --- | --- |
| **Model** | **N** | **term** | **estimate** | **std.error** | **statistic** | **p.value** |
| Depressed status | 7326 | (Intercept) | -2.63 | 0.15 | -17.91 | 0.00 |
|  |  | sexfemale | 0.51 | 0.13 | 3.92 | 0.00 |
|  |  | seriesH | -0.28 | 0.20 | -1.43 | 0.16 |
|  |  | sexfemale:seriesH | 0.40 | 0.23 | 1.73 | 0.09 |
| Depression score | 7326 | (Intercept) | 2.76 | 0.20 | 13.99 | 0.00 |
|  |  | sexfemale | 0.75 | 0.20 | 3.74 | 0.00 |
|  |  | seriesH | -0.36 | 0.23 | -1.59 | 0.12 |
|  |  | sexfemale:seriesH | 0.66 | 0.29 | 2.28 | 0.03 |
|  |  |  |  |  |  |  |


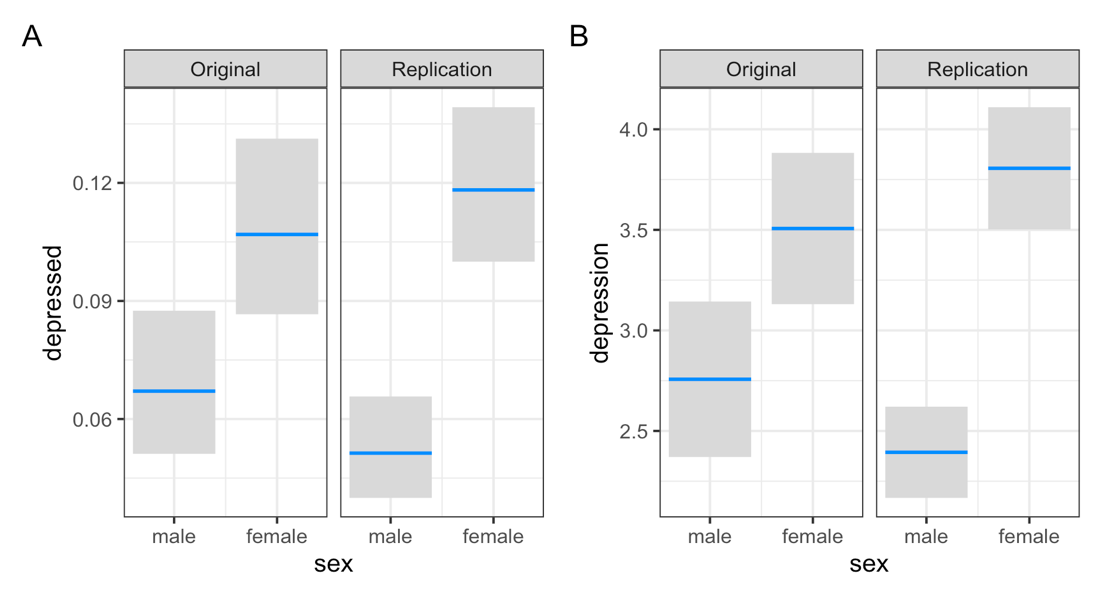


Figure S5: Effect plots of the interaction of sex and series on depressed status (proportion depressed) (A) and PHQ9 depression score (range: 0-27) (B). For model coefficients and other statistics, see Table S16.

## Interaction of sex with NHANES series in exact replication regression models

To test if the coefficient of sex differed significantly in our exact replications by NHANES data series (G or H), we fit our two exact replication models (anthropometric and socioeconomic) of depressed status and depression score with additional interaction terms between sex and series. See Table S17.

| Table S17: Regression coefficients of anthropometric and socioeconomic models of depressed status and depresion score that include an interaction term for sex with NHANES data series (G or H). | | | | | | |
| --- | --- | --- | --- | --- | --- | --- |
| **Model** | **N** | **term** | **estimate** | **std.error** | **statistic** | **p.value** |
| Anthropometric (depressed status) | 6885 | (Intercept) | -2.43 | 0.19 | -12.56 | 0.00 |
|  |  | age_centered | -0.01 | 0.12 | -0.06 | 0.95 |
|  |  | strength_centered | -0.77 | 0.25 | -3.04 | 0.01 |
|  |  | sexfemale | -0.34 | 0.21 | -1.61 | 0.12 |
|  |  | BMI_category[30,85) | 0.01 | 0.19 | 0.07 | 0.94 |
|  |  | weight_centered | 0.26 | 0.18 | 1.50 | 0.15 |
|  |  | height_centered | -0.18 | 0.23 | -0.77 | 0.45 |
|  |  | seriesH | -0.26 | 0.19 | -1.42 | 0.17 |
|  |  | age_centered:strength_centered | -0.60 | 0.19 | -3.12 | 0.01 |
|  |  | sexfemale:BMI_category[30,85) | 0.47 | 0.24 | 1.94 | 0.06 |
|  |  | sexfemale:seriesH | 0.40 | 0.22 | 1.82 | 0.08 |
| Anthropometric (depression score) | 6885 | (Intercept) | 3.23 | 0.23 | 13.92 | 0.00 |
|  |  | age_centered | -0.10 | 0.15 | -0.68 | 0.50 |
|  |  | strength_centered | -1.32 | 0.27 | -4.91 | 0.00 |
|  |  | sexfemale | -0.63 | 0.25 | -2.48 | 0.02 |
|  |  | BMI_category[30,85) | -0.34 | 0.20 | -1.68 | 0.11 |
|  |  | weight_centered | 0.80 | 0.22 | 3.56 | 0.00 |
|  |  | height_centered | -0.22 | 0.23 | -0.97 | 0.34 |
|  |  | seriesH | -0.29 | 0.20 | -1.44 | 0.16 |
|  |  | age_centered:strength_centered | -0.58 | 0.27 | -2.15 | 0.04 |
|  |  | sexfemale:BMI_category[30,85) | 1.27 | 0.30 | 4.26 | 0.00 |
|  |  | sexfemale:seriesH | 0.56 | 0.27 | 2.07 | 0.05 |
| Socioeconomic (depressed status) | 6436 | (Intercept) | -2.74 | 0.20 | -13.61 | 0.00 |
|  |  | age_centered | 0.32 | 0.15 | 2.20 | 0.04 |
|  |  | strength_centered | -0.42 | 0.23 | -1.82 | 0.08 |
|  |  | sexfemale | 0.19 | 0.26 | 0.72 | 0.48 |
|  |  | income_centered | -1.03 | 0.21 | -4.92 | 0.00 |
|  |  | edu_centered | -0.54 | 0.20 | -2.75 | 0.01 |
|  |  | living_alone | 0.40 | 0.19 | 2.05 | 0.05 |
|  |  | seriesH | -0.30 | 0.19 | -1.58 | 0.13 |
|  |  | age_centered:strength_centered | -0.59 | 0.19 | -3.14 | 0.00 |
|  |  | sexfemale:seriesH | 0.46 | 0.23 | 1.96 | 0.06 |
| Socioeconomic (depression score) | 6436 | (Intercept) | 2.91 | 0.20 | 14.93 | 0.00 |
|  |  | age_centered | 0.44 | 0.15 | 2.85 | 0.01 |
|  |  | strength_centered | -0.75 | 0.20 | -3.67 | 0.00 |
|  |  | sexfemale | 0.18 | 0.27 | 0.68 | 0.51 |
|  |  | income_centered | -1.53 | 0.18 | -8.48 | 0.00 |
|  |  | edu_centered | -0.68 | 0.22 | -3.03 | 0.01 |
|  |  | living_alone | 0.60 | 0.24 | 2.53 | 0.02 |
|  |  | seriesH | -0.28 | 0.20 | -1.43 | 0.17 |
|  |  | age_centered:strength_centered | -0.71 | 0.24 | -2.92 | 0.01 |
|  |  | sexfemale:seriesH | 0.60 | 0.28 | 2.15 | 0.04 |

## Socioeconomic model with race


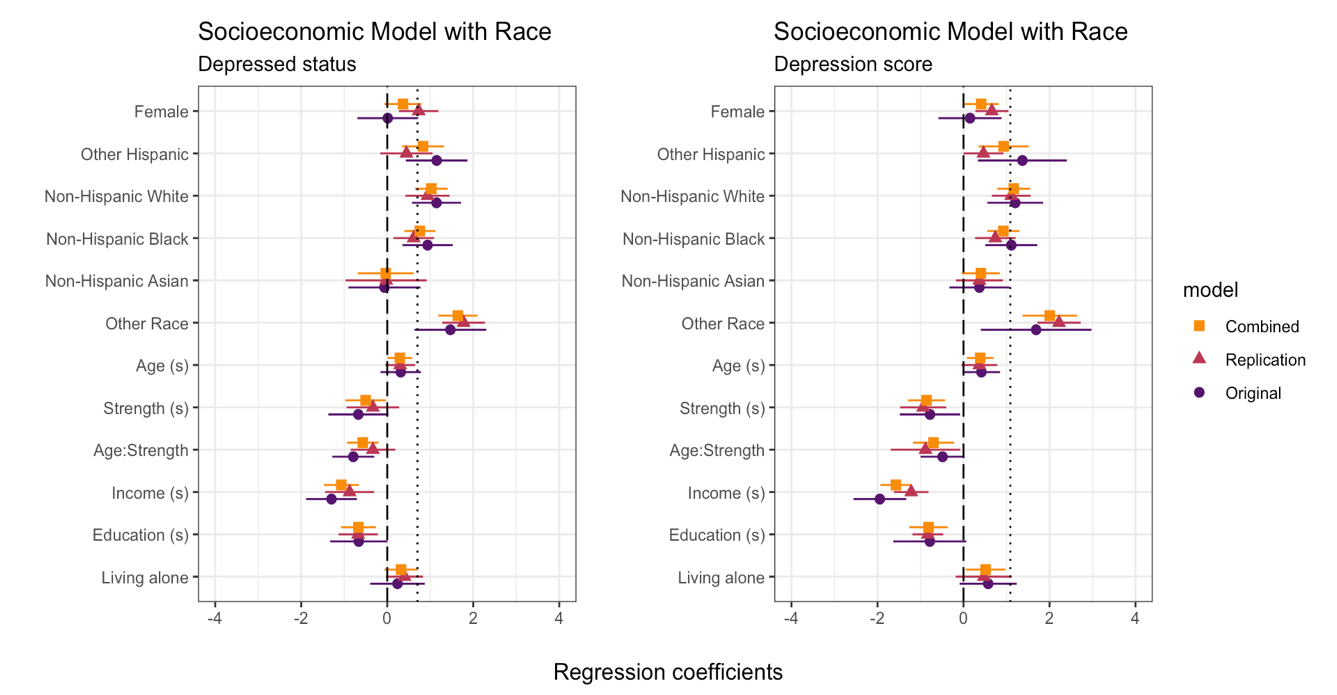


Figure S6: Coefficient plots for the Socioeconomic models of Depressed Status and Depression Score that include race. Variables with (s) have been centered at their means and standardized by 2 SD. Dotted line marks the coefficient of sex alone.

| Table S18: Regression coefficients of disability models of depressed status (quasi-binomial). | | | | | | |
| --- | --- | --- | --- | --- | --- | --- |
| **Model** | **N** | **term** | **estimate** | **std.error** | **statistic** | **p.value** |
| Original | 3033 | (Intercept) | -3.73 | 0.44 | -8.46 | 0.00 |
|  |  | age_centered | 0.31 | 0.24 | 1.31 | 0.25 |
|  |  | strength_centered | -0.67 | 0.36 | -1.89 | 0.12 |
|  |  | sexfemale | 0.01 | 0.36 | 0.03 | 0.97 |
|  |  | raceOtherHispanic | 1.15 | 0.37 | 3.14 | 0.03 |
|  |  | raceNonHispanicWhite | 1.15 | 0.29 | 3.94 | 0.01 |
|  |  | raceNonHispanicBlack | 0.94 | 0.30 | 3.14 | 0.03 |
|  |  | raceNonHispanicAsian | -0.06 | 0.43 | -0.15 | 0.89 |
|  |  | raceOtherRace | 1.47 | 0.43 | 3.45 | 0.02 |
|  |  | income_centered | -1.30 | 0.30 | -4.31 | 0.01 |
|  |  | edu_centered | -0.66 | 0.34 | -1.95 | 0.11 |
|  |  | living_alone | 0.24 | 0.32 | 0.73 | 0.50 |
|  |  | age_centered:strength_centered | -0.79 | 0.25 | -3.17 | 0.02 |
| Replication | 3403 | (Intercept) | -3.85 | 0.31 | -12.27 | 0.00 |
|  |  | age_centered | 0.30 | 0.18 | 1.70 | 0.19 |
|  |  | strength_centered | -0.33 | 0.31 | -1.07 | 0.36 |
|  |  | sexfemale | 0.73 | 0.23 | 3.13 | 0.05 |
|  |  | raceOtherHispanic | 0.45 | 0.31 | 1.43 | 0.25 |
|  |  | raceNonHispanicWhite | 0.94 | 0.26 | 3.57 | 0.04 |
|  |  | raceNonHispanicBlack | 0.62 | 0.24 | 2.55 | 0.08 |
|  |  | raceNonHispanicAsian | -0.03 | 0.48 | -0.05 | 0.96 |
|  |  | raceOtherRace | 1.78 | 0.25 | 6.99 | 0.01 |
|  |  | income_centered | -0.87 | 0.29 | -3.01 | 0.06 |
|  |  | edu_centered | -0.68 | 0.23 | -2.89 | 0.06 |
|  |  | living_alone | 0.40 | 0.22 | 1.83 | 0.17 |
|  |  | age_centered:strength_centered | -0.33 | 0.27 | -1.26 | 0.30 |
| Combined | 6436 | (Intercept) | -3.76 | 0.26 | -14.18 | 0.00 |
|  |  | age_centered | 0.29 | 0.15 | 2.02 | 0.06 |
|  |  | strength_centered | -0.50 | 0.24 | -2.10 | 0.05 |
|  |  | sexfemale | 0.37 | 0.22 | 1.67 | 0.11 |
|  |  | raceOtherHispanic | 0.83 | 0.25 | 3.33 | 0.00 |
|  |  | raceNonHispanicWhite | 1.03 | 0.19 | 5.27 | 0.00 |
|  |  | raceNonHispanicBlack | 0.76 | 0.19 | 4.09 | 0.00 |
|  |  | raceNonHispanicAsian | -0.04 | 0.33 | -0.11 | 0.92 |
|  |  | raceOtherRace | 1.64 | 0.23 | 7.07 | 0.00 |
|  |  | income_centered | -1.07 | 0.21 | -5.15 | 0.00 |
|  |  | edu_centered | -0.67 | 0.21 | -3.23 | 0.00 |
|  |  | living_alone | 0.32 | 0.20 | 1.63 | 0.12 |
|  |  | age_centered:strength_centered | -0.57 | 0.19 | -3.03 | 0.01 |

| Table S19: Regression coefficients of disability models of depression score. (Gaussian) | | | | | | |
| --- | --- | --- | --- | --- | --- | --- |
| **Model** | **N** | **term** | **estimate** | **std.error** | **statistic** | **p.value** |
| Original | 3033 | (Intercept) | 1.88 | 0.40 | 4.66 | 0.01 |
|  |  | age_centered | 0.42 | 0.22 | 1.87 | 0.12 |
|  |  | strength_centered | -0.78 | 0.36 | -2.18 | 0.08 |
|  |  | sexfemale | 0.15 | 0.38 | 0.40 | 0.70 |
|  |  | raceOtherHispanic | 1.37 | 0.53 | 2.60 | 0.05 |
|  |  | raceNonHispanicWhite | 1.20 | 0.33 | 3.64 | 0.01 |
|  |  | raceNonHispanicBlack | 1.11 | 0.31 | 3.60 | 0.02 |
|  |  | raceNonHispanicAsian | 0.37 | 0.36 | 1.04 | 0.35 |
|  |  | raceOtherRace | 1.69 | 0.66 | 2.57 | 0.05 |
|  |  | income_centered | -1.95 | 0.31 | -6.20 | 0.00 |
|  |  | edu_centered | -0.78 | 0.43 | -1.81 | 0.13 |
|  |  | living_alone | 0.57 | 0.34 | 1.69 | 0.15 |
|  |  | age_centered:strength_centered | -0.49 | 0.26 | -1.86 | 0.12 |
| Replication | 3403 | (Intercept) | 1.78 | 0.20 | 8.93 | 0.00 |
|  |  | age_centered | 0.37 | 0.21 | 1.73 | 0.18 |
|  |  | strength_centered | -0.94 | 0.27 | -3.43 | 0.04 |
|  |  | sexfemale | 0.66 | 0.20 | 3.34 | 0.04 |
|  |  | raceOtherHispanic | 0.46 | 0.24 | 1.96 | 0.14 |
|  |  | raceNonHispanicWhite | 1.11 | 0.23 | 4.83 | 0.02 |
|  |  | raceNonHispanicBlack | 0.74 | 0.24 | 3.07 | 0.05 |
|  |  | raceNonHispanicAsian | 0.37 | 0.28 | 1.32 | 0.28 |
|  |  | raceOtherRace | 2.22 | 0.26 | 8.66 | 0.00 |
|  |  | income_centered | -1.22 | 0.21 | -5.91 | 0.01 |
|  |  | edu_centered | -0.83 | 0.18 | -4.54 | 0.02 |
|  |  | living_alone | 0.47 | 0.33 | 1.42 | 0.25 |
|  |  | age_centered:strength_centered | -0.88 | 0.41 | -2.14 | 0.12 |
| Combined | 6436 | (Intercept) | 1.82 | 0.21 | 8.60 | 0.00 |
|  |  | age_centered | 0.39 | 0.16 | 2.45 | 0.02 |
|  |  | strength_centered | -0.86 | 0.22 | -3.89 | 0.00 |
|  |  | sexfemale | 0.41 | 0.21 | 1.96 | 0.06 |
|  |  | raceOtherHispanic | 0.93 | 0.30 | 3.13 | 0.01 |
|  |  | raceNonHispanicWhite | 1.17 | 0.20 | 5.99 | 0.00 |
|  |  | raceNonHispanicBlack | 0.93 | 0.19 | 4.87 | 0.00 |
|  |  | raceNonHispanicAsian | 0.40 | 0.23 | 1.78 | 0.09 |
|  |  | raceOtherRace | 2.01 | 0.32 | 6.18 | 0.00 |
|  |  | income_centered | -1.57 | 0.19 | -8.50 | 0.00 |
|  |  | edu_centered | -0.81 | 0.23 | -3.57 | 0.00 |
|  |  | living_alone | 0.51 | 0.24 | 2.16 | 0.04 |
|  |  | age_centered:strength_centered | -0.70 | 0.24 | -2.86 | 0.01 |

# 
